# Supplementary material for: A taxon-restricted duplicate of Iroquois3 is required for patterning the spider waist
Source: PLoS Biol. 2024 Aug 29;22(8):e3002771. doi: 10.1371/journal.pbio.3002771 (PMC11361693; doi:10.1371/journal.pbio.3002771)
Supplement: S1 Table — (DOCX) [file pbio.3002771.s005.docx]

**Table S1.** List of genes screened via RNAi in *Parasteatoda tepidariorum*. Gene names of uncharacterized transcripts are listed as the *P. tepidariorum* NCBI GenBank LOC numbers. Gene names and identifiers of orthologous *A. hentzi* transcripts can be found in Table S2.

| Gene | | Number injected | | Number survived to laying (C1) | | Number survived to laying (C2) | | Embryos? | Phenotype? |
| --- | --- | --- | --- | --- | --- | --- | --- | --- | --- |
| *waist-less* | | 32 | | 23 | | 22 | | Yes | Yes |
| *pnr2* | | 4 | | 3 | | 3 | | Yes | Yes |
| *biniou* | | 12 | | 9 | | 9 | | Yes | No |
| *Hand2-2* | | 4 | | 4 | | 3 | | Yes | No |
| *Sox8* | | 7 | | 7 | | 6 | | Yes | No |
| *piopio* | | 4 | | 3 | | 3 | | Yes | No |
| *LOC107448339* | | 3 | | 2 | | 2 | | Yes | No |
| *Pax9-1* | | 3 | | 2 | | 2 | | Yes | No |
| *SDPEF* | 3 | | 0 | | 0 | | Yes | | No |
| *spaetzle* | | 3 | | 0 | | 0 | | Yes | No |
| *LOC107451820* | | 3 | | 0 | | 0 | | Yes | No |
| *Mab21-1* | | 4 | | 4 | | 1 | | Yes | No |
