# Supplementary material for: A taxon-restricted duplicate of Iroquois3 is required for patterning the spider waist
Source: PLoS Biol. 2024 Aug 29;22(8):e3002771. doi: 10.1371/journal.pbio.3002771 (PMC11361693; doi:10.1371/journal.pbio.3002771)
Supplement: S2 Table — (DOCX) [file pbio.3002771.s006.docx]

**Table S2.** Identifying information for candidate gene orthologs in the tarantula *A. hentzi* and the house spider *P. tepidariorum*.

| *A. hentzi* gene ID used in this study (Ontano et al. 2021 transcriptome) | Gene nomenclature used in this study | *P. tepidariorum* ortholog (NCBI) | Alternative *A. hentzi* gene ID (Ballesteros, Setton, Santibáñez-López et al. 2020 transcriptome) |
| --- | --- | --- | --- |
|  |  |  |  |
| TRINITY_DN3405_c0_g1_i1 | *waist-less* | LOC107456088 (XM_016073862.2) | TRINITY_DN92247_c2_g2 |
| TRINITY_DN6461_c1_g1_i1 | *Mab21-1* | LOC107443737 (XM_016057696.1) | TRINITY_DN99563_c10_g1 |
| TRINITY_DN6014_c0_g1_i1 | *Sox8* | LOC107444744 (XM_016058966.1) | TRINITY_DN99433_c0_g2 |
| TRINITY_DN6222_c0_g1_i1 | *TRINITY_DN6222_c0_g1* or *LOC107451820* (species specific) | LOC107451820 (XM_016068055.2) | TRINITY_DN95745_c0_g1 |
| TRINITY_DN15528_c0_g1_i4 | *Pax9-1* | LOC107441569 (XM_016054880.1) | TRINITY_DN93988_c0_g1 |
| TRINITY_DN4873_c0_g1_i2 | *SPDEF* (*SAM pointed domain containing ETS transcription factor*) | LOC107451717 (XM_016067906.1) | TRINITY_DN99384_c3_g3 |
| TRINITY_DN18487_c0_g3_i1 | *spaetzle* | LOC107439507 (XM_016052139.1) | TRINITY_DN92783_c0_g1 |
| TRINITY_DN3695_c1_g1_i1 | *TRINITY_DN3695_c1_g1* or *LOC107448339* (species specific) | LOC107448339 (XM_016063495.1) | TRINITY_DN101009_c1_g2 |
| TRINITY_DN11929_c0_g2_i3 | *biniou* | LOC107456534 (XM_016074416.1) | TRINITY_DN100074_c0_g1 |
| TRINITY_DN5346_c1_g1_i1 | *pannier2* | LOC107439679 (XM_016052354.2) | TRINITY_DN95685_c3_g3 |
| TRINITY_DN1838_c0_g1_i1 | *Hand2-2* | LOC107443767 (XM_016057736.1) | TRINITY_DN91475_c0_g1 |
| TRINITY_DN57614_c0_g1_i1 | *piopio* | LOC107437037 (XM_016048904.1) | TRINITY_DN101436_c5_g1 |
