# Supplementary material for: A taxon-restricted duplicate of Iroquois3 is required for patterning the spider waist
Source: PLoS Biol. 2024 Aug 29;22(8):e3002771. doi: 10.1371/journal.pbio.3002771 (PMC11361693; doi:10.1371/journal.pbio.3002771)
Supplement: S3 Table — (DOCX) [file pbio.3002771.s007.docx]

**Table S3.** List of genomes referenced for synteny analysis.

| **Species** | **Taxon** | **GenBank asembly** |
| --- | --- | --- |
| *Ixodes scapularis* | apulmonate arachnids | GCF_016920785.2 |
| *Phalangium opilio* | apulmonate arachnids | GCA_019434445.1 |
| *Dysdera silvatica* | Arachnopulmonata | GCA_006491805.2 |
| *Parasteatoda tepidariorum* | Arachnopulmonata | GCF_000365465.3 |
| *Glomeris maerens* | Myriapoda | GCA_023279145.1 |
| *Rhysida immarginata* | Myriapoda | GCA_023313115.1 |
| *Thereuonema tuberculata* | Myriapoda | GCA_023159025.1 |
| *Niponia nodulosa* | Myriapoda | GCA_023159045.1 |
| *Eriocheir sinensis* | crustaceans | GCF_024679095.1 |
| *Daphnia magna* | crustaceans | GCF_020631705.1 |
| *Drosophila melanogaser* | Hexapoda | GCF_000001215.4 |
| *Tribolium castaneum* | Hexapoda | GCF_000002335.3 |
| *Epiperipatus broadwayi* | Onychophora | GCA_028023455.1 |
| *Ramazzottius varieornatus* | Tardigrada | GCA_001949185.1 |
