# Supplementary material for: A taxon-restricted duplicate of Iroquois3 is required for patterning the spider waist
Source: PLoS Biol. 2024 Aug 29;22(8):e3002771. doi: 10.1371/journal.pbio.3002771 (PMC11361693; doi:10.1371/journal.pbio.3002771)
Supplement: S4 Table — (DOCX) [file pbio.3002771.s008.docx]

**Table S4.** List of primer sequences used for gene cloning and/or riboprobe synthesis.

| **Primers** |  |  |  |  |  |
| --- | --- | --- | --- | --- | --- |
| All primers ordered with T7 linker sequences: | | |  |  |  |
| Forward primer:    ggccgcgg | |  |  |  |  |
| Reverse primer:    cccggggc | |  |  |  |  |
|  |  |  |  |  |  |
| Genes with Phenotypes | |  |  |  |  |
| **Species** | **Gene** | **Forward Primer** | **Reverse Primer** | **Length (bp)** | ***P. tepidariorum* source sequence** |
| *P. tepidariorum* | *waist-less* | CGTACGGAGATCGGTTTGAT | CCCATTTGGGCATAAAATTG | 978 | XM_016073862.1 |
| *P. tepidariorum* | *waist-less* 5' fragment | CGTACGGAGATCGGTTTGAT | TTCAGGGAATTTGGTTTCTGA | 473 | XM_016073862.1 |
| *P. tepidariorum* | *waist-less* 3' fragment | AGACTGCCACCTCAGAGAGC | CCCCATTGGGCATAAAATTG | 405 | XM_016073862.1 |
| *P. tepidariorum* | *pannier2* | ATGGACATGGAACTGCACAA | AAGTAGGCGGTGACGAAGAA | 842 | XM_016052354.2 |
|  |  |  |  |  |  |
| Genes used for expression assays | |  |  |  |  |
| **Species** | **Gene** | **Forward Primer** | **Reverse Primer** | **Length (bp)** | ***P. tepidariorum* source sequence** |
| *P. tepidariorum* | *Scr-1* | AATTGCGAGGTTGTTCTTC | CAGACACAGGGCATGAGCTA | 750 | FM956097.1 |
| *P. tepidariorum* | *Dll* | ATGCCCAGGCTTACCCTATT | TGTCCCATGAGGAGATAGGC | 819 | FM876233.2 |
| *P. tepidariorum* | *en* | CTGCTTGACATTGCCTGAAA | GAATCTGCTGGCATTCCATT | 792 | AB125741.1 |
|  |  |  |  |  |  |
| Genes resulting in no phenotype | |  |  |  |  |
|  |  |  |  |  |  |
| **Species** | **Gene** | **Forward Primer** | **Reverse Primer** | **Length (bp)** | ***P. tepidariorum* source sequence** |
| *P. tepidariorum* | *biniou* | AAGGCCAGGAAAAGGACATT | ACTGTTTCGGAGGTCACAGG | 803 | XM_016074416.1 |
| *P. tepidariorum* | *Hand2-2* | GGTCCTGGTGGTGAAACATT | ACGGGACCAACTCGTAATCA | 799 | XM_016061345.1 |
| *P. tepidariorum* | *Mab21-1* | ATGGACATTTTGACGGCTTC | CGCGGATTTTCCTTTAAACA | 778 | XM_016057696.1 |
| *P. tepidariorum* | *Pax9-1* | CCTGCGACATCTCAAGACAG | AACATTTCCTGTTGTCGCCTA | 790 | XM_016054880.1 |
| *P. tepidariorum* | *SAM pointed domain-containing Ets transcription factor* | ACAATGGCCGATTTTGAGAG | CAACGTCACAGGATTCATCG | 858 | XM_016067906.1 |
| *P. tepidariorum* | *piopio* | TCATGTTCTCCACCCAGTCA | TCTTGAGGCACAGTTTGACG | 763 | XM_016048904.1 |
| *P. tepidariorum* | *Sox8* | GTGGGGGTTCTCCCAGTAAT | AACATAAGGGTCTGCCGTTG | 859 | XM_016058966.1 |
| *P. tepidariorum* | *spaetzle* | TTTGTGGGAGAAAGGGAGAA | CCAGATGCGATTTCTTTCGT | 770 | XM_016052139.1 |
| *P. tepidariorum* | *LOC107448339 uncharacterized protein* | TGCGAGTTCTTGGAATGATG | TCAGATTTTCTTAGTTCGGTGACA | 656 | XM_016063495.1 |
| *P. tepidariorum* | *LOC107451820 uncharacterized protein* | ACACGGAGAATGTGCCTACC | CAGTGAAACCAAGCGAGACA | 795 | XM_016068055.2 |
