# Supplementary material for: A taxon-restricted duplicate of Iroquois3 is required for patterning the spider waist
Source: PLoS Biol. 2024 Aug 29;22(8):e3002771. doi: 10.1371/journal.pbio.3002771 (PMC11361693; doi:10.1371/journal.pbio.3002771)
Supplement: S5 Table — (DOCX) [file pbio.3002771.s009.docx]

**Table S5.** List of HCR probe sequences.

| **Initiator and gene name** | **Sequence** |
| --- | --- |
| B2_Ptep_waist-less | AAATCTTCTAATTTCGTCCTATTGGGT |
| B2_Ptep_waist-less | TTAGCTCTCACACCGACTTGGAATAAA |
| B2_Ptep_waist-less | AAGCTGGAGCACTTGTAAAACCAACGG |
| B2_Ptep_waist-less | GATGTAGAAACTGTATGATACATAGAA |
| B2_Ptep_waist-less | AACTGGCTGAAATGTCCTTATGTCTAG |
| B2_Ptep_waist-less | ATGAAAAAGAGGATGTCCTCAAGAAAA |
| B2_Ptep_waist-less | AAATAGTCTATAGGATTATGCCTCAGA |
| B2_Ptep_waist-less | ATGTCCCATTTGGGCATAAAATTGTAA |
| B2_Ptep_waist-less | AACTACCATTCAGAAGGCGATTGTAAT |
| B2_Ptep_waist-less | TGTGAACTGTCACCACAAGGTAAAAAA |
| B2_Ptep_waist-less | AAAGGAGGATGGTGACACAGAAGCTAA |
| B2_Ptep_waist-less | TGGGTGGTAAAACGGATGGGGATCCAA |
| B2_Ptep_waist-less | AAGAATTTGTCACCATGTGGGAAATTG |
| B2_Ptep_waist-less | ACTTCGCTCGTGGGAGGTGACACTAAA |
| B2_Ptep_waist-less | AAGACGATAAAGATGATTCGGCTGAAC |
| B2_Ptep_waist-less | AACTCTCCTGTTCTCCAAAGGGGTGAA |
| B2_Ptep_waist-less | AAGTGAAGGATGGTATAAGTGTTCATT |
| B2_Ptep_waist-less | CCTGCGGAGGAAGAGGCCTGTGGTAAA |
| B2_Ptep_waist-less | AAATCAGTTTGGTGGTCGAAGAACTTT |
| B2_Ptep_waist-less | ACCCAACCTTCTTAATTTTTGGTTCAA |
| B2_Ptep_waist-less | AAGCGCTAGTGACCAGATTTTTGGTTT |
| B2_Ptep_waist-less | GTGGTGGGCTCTCTGAGGTGGCAGTAA |
| B2_Ptep_waist-less | AAAAGTTCAGGGAATTTGGTTTCTGAG |
| B2_Ptep_waist-less | AATTGATTCTGATTTGATGTGTTCAAA |
| B2_Ptep_waist-less | AATCGGGAGCACTGAGGCTGTCACCAA |
| B2_Ptep_waist-less | TCAAGCGCTTTCATTGGAGACCTGTAA |
| B2_Ptep_waist-less | AACATCGTCCTTCATACTGTCTCGTCT |
| B2_Ptep_waist-less | GGGAGGTGCATTTCTGTTGTCGGTTAA |
| B2_Ptep_waist-less | AATTGATTTTCATTCGAATCGCTGAGT |
| B2_Ptep_waist-less | TCTACTGGAGTCACTTTCCTGGCATAA |
| B2_Ptep_waist-less | AATCATCTTTATCATCATCTTCATCCA |
| B2_Ptep_waist-less | CTCTTAAGGTCATCCCCACTCTGCTAA |
| B2_Ptep_waist-less | AAATCTCCGTACGGAGAATATGCGGCG |
| B2_Ptep_waist-less | TTTTTCACTATCAACAGGATCAAACAA |
| B2_Ptep_waist-less | AAAGCCCGAGGATCTTTTATGTCATAA |
| B2_Ptep_waist-less | GTGTCCTGGTGGTAGGGATGCCCAGAA |
| B2_Ptep_waist-less | AAGTGGCATGCTGTTTGAGGCTGGATA |
| B2_Ptep_waist-less | GATGGGCCTGACTTACTAGTGTGTCAA |
| B2_Ptep_waist-less | AATTGAGGAGATGCAGTATAGGCGAAA |
| B2_Ptep_waist-less | GCTGGGGTGCTGGCCTGTCATCAGAAA |
|  |  |
|  |  |
| **Initiator and gene name** | **Sequence** |
| B1_Ptep_pnr-2 | GAGGAGGGCAGCAAACGGAAGTATGGCGCGGGCATTCTATTTGAC |
| B1_Ptep_pnr-2 | TTAACCATCACGAATCTCATGTTTGTAGAAGAGTCTTCCTTTACG |
| B1_Ptep_pnr-2 | GAGGAGGGCAGCAAACGGAAGTCCATGCTGTGATGACTGTTATAG |
| B1_Ptep_pnr-2 | TTGGTGGTGATGGCTTGAACTGGAGTAGAAGAGTCTTCCTTTACG |
| B1_Ptep_pnr-2 | GAGGAGGGCAGCAAACGGAAAGCAGGGGTTGAATTGTTGCTTACT |
| B1_Ptep_pnr-2 | AGTTGCATCGCAAGATGCTGGAGAATAGAAGAGTCTTCCTTTACG |
| B1_Ptep_pnr-2 | GAGGAGGGCAGCAAACGGAATGGTGGTCTGTTTACCCCATGTAAT |
| B1_Ptep_pnr-2 | TTTTTGAATTCCTTCCTTTTTCATTTAGAAGAGTCTTCCTTTACG |
| B1_Ptep_pnr-2 | GAGGAGGGCAGCAAACGGAACAGATATTGCCGGATCTAGCAATCC |
| B1_Ptep_pnr-2 | CATCAGAACAATATGGATGTTGCAATAGAAGAGTCTTCCTTTACG |
| B1_Ptep_pnr-2 | GAGGAGGGCAGCAAACGGAAGTCAAAGCTGAAATTCCATAACCAG |
| B1_Ptep_pnr-2 | GAATTTTGAGCAAGATTCAGTCCAGTAGAAGAGTCTTCCTTTACG |
| B1_Ptep_pnr-2 | GAGGAGGGCAGCAAACGGAAAAGCAGCGCTGGAGGGAGATGATGG |
| B1_Ptep_pnr-2 | CGAGACTTCCAGTTGACGAAGCATATAGAAGAGTCTTCCTTTACG |
| B1_Ptep_pnr-2 | GAGGAGGGCAGCAAACGGAAATAGTTGGAGCACGTTCCCTGTTTC |
| B1_Ptep_pnr-2 | ACCATGACTGTATCACTGGAACTGCTAGAAGAGTCTTCCTTTACG |
| B1_Ptep_pnr-2 | GAGGAGGGCAGCAAACGGAAGTCGACTGTTCTCTATGATTGATGG |
| B1_Ptep_pnr-2 | CGACTATTAGATGTTTCATTGGCTGTAGAAGAGTCTTCCTTTACG |
| B1_Ptep_pnr-2 | GAGGAGGGCAGCAAACGGAATCATTGCGCAAGAAGAAGTATTCTC |
| B1_Ptep_pnr-2 | TTGCTAAGCTGTTGTTGTTATTCGATAGAAGAGTCTTCCTTTACG |
| B1_Ptep_pnr-2 | GAGGAGGGCAGCAAACGGAAAGCATGTTCTGTAACATTTGCATGG |
| B1_Ptep_pnr-2 | GTTACAATGATATGAATCTGAGACGTAGAAGAGTCTTCCTTTACG |
| B1_Ptep_pnr-2 | GAGGAGGGCAGCAAACGGAATGCTTTGATGGTCAGTTTTGGACAG |
| B1_Ptep_pnr-2 | TAGCATGGTGGTGTGAATCCCATTCTAGAAGAGTCTTCCTTTACG |
| B1_Ptep_pnr-2 | GAGGAGGGCAGCAAACGGAACAGTTCCATGTCCATCGTTTCTATT |
| B1_Ptep_pnr-2 | TTTTTGCCATTTGAGTAGTTTCTTGTAGAAGAGTCTTCCTTTACG |
| B1_Ptep_pnr-2 | GAGGAGGGCAGCAAACGGAATAATCTTCAACAGCGAGAACATTTG |
| B1_Ptep_pnr-2 | TTTTTGACTGCTGGAGGAACTAAACTAGAAGAGTCTTCCTTTACG |
| B1_Ptep_pnr-2 | GAGGAGGGCAGCAAACGGAAATTACTGTCATCTCTTGATACGGTT |
| B1_Ptep_pnr-2 | TTGTACTGCTTTATTCACTCCCGCGTAGAAGAGTCTTCCTTTACG |
| B1_Ptep_pnr-2 | GAGGAGGGCAGCAAACGGAAAAGATTCTTTGGGAAGAACGGGCTG |
| B1_Ptep_pnr-2 | TACCAAAATCATTTACATTACTGTCTAGAAGAGTCTTCCTTTACG |
| B1_Ptep_pnr-2 | GAGGAGGGCAGCAAACGGAAAATGTGCTGGCTCCAGCACTAAATA |
| B1_Ptep_pnr-2 | GTATAGAATTTTTGGTCATAGAATTTAGAAGAGTCTTCCTTTACG |
| B1_Ptep_pnr-2 | GAGGAGGGCAGCAAACGGAAAAATTATGGGCAGAAGTAAATACGG |
| B1_Ptep_pnr-2 | TGTGCTTCATGATTGTTAACTAACGTAGAAGAGTCTTCCTTTACG |
| B1_Ptep_pnr-2 | GAGGAGGGCAGCAAACGGAACTAAAGTTTCTGGCTTCATATTGCG |
| B1_Ptep_pnr-2 | CAAAGTTCTTATAAGACAAGTTGCGTAGAAGAGTCTTCCTTTACG |
| B1_Ptep_pnr-2 | GAGGAGGGCAGCAAACGGAACTTTCGACGATTTGAGGATAGGATT |
| B1_Ptep_pnr-2 | ATGCTGTTTAAATTCTTCGAAGCTTTAGAAGAGTCTTCCTTTACG |
| B1_Ptep_pnr-2 | GAGGAGGGCAGCAAACGGAACTTTCGACGATTTGAGGATAGGATT |
| B1_Ptep_pnr-2 | ATGCTGTTTAAATTCTTCGAAGCTTTAGAAGAGTCTTCCTTTACG |
| B1_Ptep_pnr-2 | GAGGAGGGCAGCAAACGGAACTTTCGACGATTTGAGGATAGGATT |
| B1_Ptep_pnr-2 | ATGCTGTTTAAATTCTTCGAAGCTTTAGAAGAGTCTTCCTTTACG |
| B1_Ptep_pnr-2 | GAGGAGGGCAGCAAACGGAACTTTCGACGATTTGAGGATAGGATT |
| B1_Ptep_pnr-2 | ATGCTGTTTAAATTCTTCGAAGCTTTAGAAGAGTCTTCCTTTACG |
|  |  |
|  |  |
| **Initiator and gene name** | **Sequence** |
| B3_Ptep_sog | GTCCCTGCCTCTATATCTTTGTGTATAAAAGCATAGCACCACCGA |
| B3_Ptep_sog | TTTTTGTTATAAAATGGGTAGCCCATTCCACTCAACTTTAACCCG |
| B3_Ptep_sog | GTCCCTGCCTCTATATCTTTCTCAGTTTTGTTTCTCTTTGGGTTG |
| B3_Ptep_sog | CAACAAATGCCTGTTGGATGATGGATTCCACTCAACTTTAACCCG |
| B3_Ptep_sog | GTCCCTGCCTCTATATCTTTGACACAGAATCTATGCATCTATCAC |
| B3_Ptep_sog | CCTTCCGCGGATCTTGAAGACGATTTTCCACTCAACTTTAACCCG |
| B3_Ptep_sog | GTCCCTGCCTCTATATCTTTATGACATTTAACACATTTTTCAGCT |
| B3_Ptep_sog | CTTACACTTGGCTCGTCCGTCTTTATTCCACTCAACTTTAACCCG |
| B3_Ptep_sog | GTCCCTGCCTCTATATCTTTTAACAGTTGATTTTTCACCACTTTG |
| B3_Ptep_sog | TGCAACCTCCAGCATTCAATATCTCTTCCACTCAACTTTAACCCG |
| B3_Ptep_sog | GTCCCTGCCTCTATATCTTTCTATAAGCATCTTTCTCAGCACAAG |
| B3_Ptep_sog | TTTTTGCAACAATCGTTATCTTGCTTTCCACTCAACTTTAACCCG |
| B3_Ptep_sog | GTCCCTGCCTCTATATCTTTGGGAGGAACATAGGGATGCCATCTG |
| B3_Ptep_sog | ACAAAGAGAGCATTTGCTGAATCCATTCCACTCAACTTTAACCCG |
| B3_Ptep_sog | GTCCCTGCCTCTATATCTTTAACATTCTCCTTCCATCGTCATAGG |
| B3_Ptep_sog | CATTAGAGTTACTTGGGCAGAAGGGTTCCACTCAACTTTAACCCG |
| B3_Ptep_sog | GTCCCTGCCTCTATATCTTTTCACCTTTCCAATGATCTCCATCAT |
| B3_Ptep_sog | CAAGAGCACATGGTGCATTCTTCATTTCCACTCAACTTTAACCCG |
| B3_Ptep_sog | GTCCCTGCCTCTATATCTTTTCTTGTCTTGGAGGATGCATCGTAA |
| B3_Ptep_sog | GTATCCATCCTGATATGTATTCCCATTCCACTCAACTTTAACCCG |
| B3_Ptep_sog | GTCCCTGCCTCTATATCTTTAGTGTAGCCTTGTTGTATGAAAGCC |
| B3_Ptep_sog | GTTAAGTTTCGATGTCACAACCAGGTTCCACTCAACTTTAACCCG |
| B3_Ptep_sog | GTCCCTGCCTCTATATCTTTTGATATTTTCCTGGTCTCACTTGCA |
| B3_Ptep_sog | TTTTTCAAGACACGCTGTAGAGGGTTTCCACTCAACTTTAACCCG |
| B3_Ptep_sog | GTCCCTGCCTCTATATCTTTCCAAGCTATTCCACCTGCAGTTGTG |
| B3_Ptep_sog | AAGAATGCAATCTTTATCAATGGATTTCCACTCAACTTTAACCCG |
| B3_Ptep_sog | GTCCCTGCCTCTATATCTTTGAACTTTGTCAGAACGTGTAACACG |
| B3_Ptep_sog | GATGAACTTGGCCTCGCAACTGCATTTCCACTCAACTTTAACCCG |
| B3_Ptep_sog | GTCCCTGCCTCTATATCTTTACTGTATTAGGTGTGTCTGCAAGTA |
| B3_Ptep_sog | CGATATTCTGGAGTAGCTGAATGTATTCCACTCAACTTTAACCCG |
| B3_Ptep_sog | GTCCCTGCCTCTATATCTTTTAGGTTTTGTGAAACTTGTTGTAAC |
| B3_Ptep_sog | GAATGTGCCATTTCCCCATGAATTATTCCACTCAACTTTAACCCG |
| B3_Ptep_sog | GTCCCTGCCTCTATATCTTTACTTATAATTTATGAATCCTTCTGA |
| B3_Ptep_sog | ATTCAATGCTTGGATCGCTGACGTGTTCCACTCAACTTTAACCCG |
| B3_Ptep_sog | GTCCCTGCCTCTATATCTTTCATGATGCGTCCGCTCTGGAGGCGT |
| B3_Ptep_sog | TTCAAACACGTTGCAAGTAACCTTGTTCCACTCAACTTTAACCCG |
| B3_Ptep_sog | GTCCCTGCCTCTATATCTTTATCAATGGCACATCTCGGATATCTT |
| B3_Ptep_sog | TCCATATCTTCATAGAAAAGGCTAATTCCACTCAACTTTAACCCG |
| B3_Ptep_sog | GTCCCTGCCTCTATATCTTTAGTTTCATTCGCCAAATCGCCAGAT |
| B3_Ptep_sog | AATGTTCGCCATACCACCAGTACCTTTCCACTCAACTTTAACCCG |
| B3_Ptep_sog | GTCCCTGCCTCTATATCTTTTGTGAGCTAGTTACCAATATAAAGA |
| B3_Ptep_sog | ATTCTCCCTCCAACAATTCCATCAGTTCCACTCAACTTTAACCCG |
| B3_Ptep_sog | GTCCCTGCCTCTATATCTTTGGGTGCAATGGGAATTTCATGTTCT |
| B3_Ptep_sog | ACAAACTTTTGATCCTTGAACATGGTTCCACTCAACTTTAACCCG |
| B3_Ptep_sog | GTCCCTGCCTCTATATCTTTAAAACAAAGTAACCACGTGCTGCTC |
| B3_Ptep_sog | ATGGTATAATGAAGATCTCGTTTAGTTCCACTCAACTTTAACCCG |
| B3_Ptep_sog | GTCCCTGCCTCTATATCTTTCTTCATCTTCTGATTCTAGCTTCCG |
| B3_Ptep_sog | GAGCCGTATATTCTTTCAGAATTTTTTCCACTCAACTTTAACCCG |
| B3_Ptep_sog | GTCCCTGCCTCTATATCTTTCATGTTGGTTTCGGGCAATCATTCT |
| B3_Ptep_sog | CTTTGGGGAAGTAAGACAGGATCGTTTCCACTCAACTTTAACCCG |
| B3_Ptep_sog | GTCCCTGCCTCTATATCTTTTTCTCCTTTTTCGCTGAACCGGTAT |
| B3_Ptep_sog | TATTTTTGCATCGCACTTTCGACATTTCCACTCAACTTTAACCCG |
| B3_Ptep_sog | GTCCCTGCCTCTATATCTTTAAATGGAGGACCCAAGTCAGGCCTC |
| B3_Ptep_sog | TTCGCATCGCATGCAATACAGCATGTTCCACTCAACTTTAACCCG |
| B3_Ptep_sog | GTCCCTGCCTCTATATCTTTCCAAACTGACAATGAGTTTGTTTTT |
| B3_Ptep_sog | CTCTCCTCCAGTTCGTAAGTATTGTTTCCACTCAACTTTAACCCG |
| B3_Ptep_sog | GTCCCTGCCTCTATATCTTTATTCATTTTTGAATAGAGTTGCTTC |
| B3_Ptep_sog | CAATCCACACACACTTTGTTTCCGTTTCCACTCAACTTTAACCCG |
| B3_Ptep_sog | GTCCCTGCCTCTATATCTTTTCTAACAACTAAAAACATACGGCAC |
| B3_Ptep_sog | GTTTTCCCCTAGATTTTCTTTCGCATTCCACTCAACTTTAACCCG |
|  |  |
|  |  |
| **Initiator and gene name** | **Sequence** |
| B1_Ptep_Irx1_multiplexed | GAGGAGGGCAGCAAACGGAATCCAGCGCTTATGACAGTGCTACCC |
| B1_Ptep_Irx1_multiplexed | TTATGATAAAACACGACCTTGTGTCTAGAAGAGTCTTCCTTTACG |
| B1_Ptep_Irx1_multiplexed | GAGGAGGGCAGCAAACGGAAGAGGTTAGGTTGAACACTGAGGGAG |
| B1_Ptep_Irx1_multiplexed | GGTTGCAGAGAGTTGTTGCTCAGTTTAGAAGAGTCTTCCTTTACG |
| B1_Ptep_Irx1_multiplexed | GAGGAGGGCAGCAAACGGAACCGTAATCTATTCAGTGAGGACACT |
| B1_Ptep_Irx1_multiplexed | AGCAGTCGGAAAGGCCGCTGCAGCTTAGAAGAGTCTTCCTTTACG |
| B1_Ptep_Irx1_multiplexed | GAGGAGGGCAGCAAACGGAAGGATCCTCCGACACTGGTCACAACA |
| B1_Ptep_Irx1_multiplexed | ACACGAGTAAACGGCCGACAGAGGATAGAAGAGTCTTCCTTTACG |
| B1_Ptep_Irx1_multiplexed | GAGGAGGGCAGCAAACGGAAAGCACCAGGCGGTAGAGCAGACGAA |
| B1_Ptep_Irx1_multiplexed | TCTCTGAAGAGGACTCAGTCTGGAATAGAAGAGTCTTCCTTTACG |
| B1_Ptep_Irx1_multiplexed | GAGGAGGGCAGCAAACGGAAACAATGGACCATATTCGAGGTTTTT |
| B1_Ptep_Irx1_multiplexed | GGCCCTTGTGTTGACGTAGCCGTGTTAGAAGAGTCTTCCTTTACG |
| B1_Ptep_Irx1_multiplexed | GAGGAGGGCAGCAAACGGAATCGAAGCTGGGTGGGGTAGTGAAGA |
| B1_Ptep_Irx1_multiplexed | GCTGGCTACTTTGTGGTGGTAGTTCTAGAAGAGTCTTCCTTTACG |
| B1_Ptep_Irx1_multiplexed | GAGGAGGGCAGCAAACGGAACAATGAGGCATCAGAGATAGGACTT |
| B1_Ptep_Irx1_multiplexed | CTGCGAGGATTCAGAGTCTTCTGATTAGAAGAGTCTTCCTTTACG |
| B1_Ptep_Irx1_multiplexed | GAGGAGGGCAGCAAACGGAATTCTCTTTGTCGGAGCTGTTTGTGA |
| B1_Ptep_Irx1_multiplexed | ACACGATGCGTCTTCAGGAGGGAACTAGAAGAGTCTTCCTTTACG |
| B1_Ptep_Irx1_multiplexed | GAGGAGGGCAGCAAACGGAAACGACTTGCAGTCATCATCCACGTC |
| B1_Ptep_Irx1_multiplexed | TGTGGCCGTCTGTACTCGTAGATGCTAGAAGAGTCTTCCTTTACG |
| B1_Ptep_Irx1_multiplexed | GAGGAGGGCAGCAAACGGAATTGCCTAGGATGGTGATGATCGTCC |
| B1_Ptep_Irx1_multiplexed | GAAATCCATCTTACCATCGTCGGAATAGAAGAGTCTTCCTTTACG |
| B1_Ptep_Irx1_multiplexed | GAGGAGGGCAGCAAACGGAAGATCTTTTACCATCGGACTCTTTTA |
| B1_Ptep_Irx1_multiplexed | CTCCGATCTACATCGGAATCTTTATTAGAAGAGTCTTCCTTTACG |
| B1_Ptep_Irx1_multiplexed | GAGGAGGGCAGCAAACGGAACCGTTTGGAAAGGGATAACCAGCAA |
| B1_Ptep_Irx1_multiplexed | TTTTTACCGTTTAAATCAACACCATTAGAAGAGTCTTCCTTTACG |
| B1_Ptep_Irx1_multiplexed | GAGGAGGGCAGCAAACGGAAAAGCTGCATATGGTAAACTTCTCCA |
| B1_Ptep_Irx1_multiplexed | CGGCCGAATCGTATGGGTAATACATTAGAAGAGTCTTCCTTTACG |
| B1_Ptep_Irx1_multiplexed | GAGGAGGGCAGCAAACGGAAATTAGCCGTTGGAGAATAGAATGCG |
| B1_Ptep_Irx1_multiplexed | TTCTAGGTTGTCCTTTAGGTCAAAGTAGAAGAGTCTTCCTTTACG |
| B1_Ptep_Irx1_multiplexed | GAGGAGGGCAGCAAACGGAAGGGTGGTTGCAGTTGATCCGTTATA |
| B1_Ptep_Irx1_multiplexed | TTCCGGACTGTCCAGCCATCAGCAGTAGAAGAGTCTTCCTTTACG |
| B1_Ptep_Irx1_multiplexed | GAGGAGGGCAGCAAACGGAAAGATGGACAGTCATACGTATGGATG |
| B1_Ptep_Irx1_multiplexed | AAATTGGGGACATGACATTTTCAAGTAGAAGAGTCTTCCTTTACG |
| B1_Ptep_Irx1_multiplexed | GAGGAGGGCAGCAAACGGAATAGAAGTCCATACAATGTGTCTCTC |
| B1_Ptep_Irx1_multiplexed | GGTCGTCCGGTTCTCCAGGAAATCCTAGAAGAGTCTTCCTTTACG |
| B1_Ptep_Irx1_multiplexed | GAGGAGGGCAGCAAACGGAATCGTTTCATTAAGCCATGCAATTAC |
| B1_Ptep_Irx1_multiplexed | TTACCACACTAGGTTTCTGAAGTCATAGAAGAGTCTTCCTTTACG |
| B1_Ptep_Irx1_multiplexed | GAGGAGGGCAGCAAACGGAATATTTGAATTCCAAACTATAAGACC |
| B1_Ptep_Irx1_multiplexed | TTGCGTCGAAATAAACGAATCGGAATAGAAGAGTCTTCCTTTACG |
|  |  |
| B2_Ptep_Irx3-1_multiplexed | CCTCGTAAATCCTCATCAAATAACGAGGCGGAATGTTCGAGAACC |
| B2_Ptep_Irx3-1_multiplexed | TTATTCACTATGTTTAACATTCGTTAAATCATCCAGTAAACCGCC |
| B2_Ptep_Irx3-1_multiplexed | CCTCGTAAATCCTCATCAAAAATCCGTTCGAATCGAAAAATTCCG |
| B2_Ptep_Irx3-1_multiplexed | TGACAGCACTTTTGAAGTGTTCTATAAATCATCCAGTAAACCGCC |
| B2_Ptep_Irx3-1_multiplexed | CCTCGTAAATCCTCATCAAATGATTTGTTGTCATACTCTAAACAG |
| B2_Ptep_Irx3-1_multiplexed | TGTGTCACAATCCGCTTCGTAAACTAAATCATCCAGTAAACCGCC |
| B2_Ptep_Irx3-1_multiplexed | CCTCGTAAATCCTCATCAAATTCTGAGGCACACTTTATATCATTC |
| B2_Ptep_Irx3-1_multiplexed | GGGGTTGTCCATCATGCACGATCCTAAATCATCCAGTAAACCGCC |
| B2_Ptep_Irx3-1_multiplexed | CCTCGTAAATCCTCATCAAAGGCATGAAATCTGTAAAAGAGGACA |
| B2_Ptep_Irx3-1_multiplexed | TTTTTATTGCAACACAACTCACTGTAAATCATCCAGTAAACCGCC |
| B2_Ptep_Irx3-1_multiplexed | CCTCGTAAATCCTCATCAAAGGACATTTTCTAAACAGAAATCCAT |
| B2_Ptep_Irx3-1_multiplexed | TCGAGTTCACGGAACACTCTGCTTTAAATCATCCAGTAAACCGCC |
| B2_Ptep_Irx3-1_multiplexed | CCTCGTAAATCCTCATCAAACAAGCGACCAAATTTTTGGTCTGCT |
| B2_Ptep_Irx3-1_multiplexed | GGGGACTATTGGATGTGGCAGTTCTAAATCATCCAGTAAACCGCC |
| B2_Ptep_Irx3-1_multiplexed | CCTCGTAAATCCTCATCAAAGGAAACGTCTTTTGCACTAGGTTTT |
| B2_Ptep_Irx3-1_multiplexed | GGAATCGAAGGTTTCCGAAAACCTAAAATCATCCAGTAAACCGCC |
| B2_Ptep_Irx3-1_multiplexed | CCTCGTAAATCCTCATCAAATCCTAACTGGGCTAGAATGTTCTGA |
| B2_Ptep_Irx3-1_multiplexed | CCTGCTGGAATCCCGGGTAGCAGGAAAATCATCCAGTAAACCGCC |
| B2_Ptep_Irx3-1_multiplexed | CCTCGTAAATCCTCATCAAAAGTTCGATAACATTCCAGAGCACAT |
| B2_Ptep_Irx3-1_multiplexed | GATGGAATCTTCTGATGAAAGACTTAAATCATCCAGTAAACCGCC |
| B2_Ptep_Irx3-1_multiplexed | CCTCGTAAATCCTCATCAAAAAGGTTCTTTGCTGTGGAATGTTTC |
| B2_Ptep_Irx3-1_multiplexed | GGGGAGATGTGGAATAATCGGCAGGAAATCATCCAGTAAACCGCC |
| B2_Ptep_Irx3-1_multiplexed | CCTCGTAAATCCTCATCAAACTTCTGCTGTATCGTCTGACTTCAG |
| B2_Ptep_Irx3-1_multiplexed | AAAAATCGTTCAAGTTGCTGCACTCAAATCATCCAGTAAACCGCC |
| B2_Ptep_Irx3-1_multiplexed | CCTCGTAAATCCTCATCAAACTGTCTGTATAGGTTGTACAGGAGG |
| B2_Ptep_Irx3-1_multiplexed | TTTTTAACACTCTCCATAGAACCGTAAATCATCCAGTAAACCGCC |
| B2_Ptep_Irx3-1_multiplexed | CCTCGTAAATCCTCATCAAATCCATGAGTTTCGTGATTCTTTCAA |
| B2_Ptep_Irx3-1_multiplexed | TAGGATCGTATGGGTAACAGGATGCAAATCATCCAGTAAACCGCC |
| B2_Ptep_Irx3-1_multiplexed | CCTCGTAAATCCTCATCAAAAGTGGTTGTGTTGGCTGCCAGTGAT |
| B2_Ptep_Irx3-1_multiplexed | GTAAGGAGCACCCAGTGTTGAGTAGAAATCATCCAGTAAACCGCC |
| B2_Ptep_Irx3-1_multiplexed | CCTCGTAAATCCTCATCAAAAGACCGAAGGATGCCAGCCGAGGTA |
| B2_Ptep_Irx3-1_multiplexed | ATGTGATCAGCGTATGAAGAACCGTAAATCATCCAGTAAACCGCC |
| B2_Ptep_Irx3-1_multiplexed | CCTCGTAAATCCTCATCAAATACCTGTGTTGAAGAAGGTGGATAT |
| B2_Ptep_Irx3-1_multiplexed | GCAACACGGTCCGCCGCTGCTCACCAAATCATCCAGTAAACCGCC |
| B2_Ptep_Irx3-1_multiplexed | CCTCGTAAATCCTCATCAAACGATTTGGACGGATTAGTGGGGTGT |
| B2_Ptep_Irx3-1_multiplexed | TAACCGAATTGTGAATAGGACATTAAAATCATCCAGTAAACCGCC |
| B2_Ptep_Irx3-1_multiplexed | CCTCGTAAATCCTCATCAAATCATTCGGCCTTTCTTGAACCGATC |
| B2_Ptep_Irx3-1_multiplexed | GAAAGGGAGATCGGCCTCACAGTGTAAATCATCCAGTAAACCGCC |
| B2_Ptep_Irx3-1_multiplexed | CCTCGTAAATCCTCATCAAAATTCCGTTATTCCAACTGTATCCCT |
| B2_Ptep_Irx3-1_multiplexed | CAAAAACATGGCGATCATCGGTCAAAAATCATCCAGTAAACCGCC |
|  |  |
| B3_Ptep_waist-less_multiplexed | GTCCCTGCCTCTATATCTTTATCTTCTAATTTCGTCCTATTGGGT |
| B3_Ptep_waist-less_multiplexed | TTAGCTCTCACACCGACTTGGAATATTCCACTCAACTTTAACCCG |
| B3_Ptep_waist-less_multiplexed | GTCCCTGCCTCTATATCTTTGCTGGAGCACTTGTAAAACCAACGG |
| B3_Ptep_waist-less_multiplexed | GATGTAGAAACTGTATGATACATAGTTCCACTCAACTTTAACCCG |
| B3_Ptep_waist-less_multiplexed | GTCCCTGCCTCTATATCTTTCTGGCTGAAATGTCCTTATGTCTAG |
| B3_Ptep_waist-less_multiplexed | ATGAAAAAGAGGATGTCCTCAAGAATTCCACTCAACTTTAACCCG |
| B3_Ptep_waist-less_multiplexed | GTCCCTGCCTCTATATCTTTATAGTCTATAGGATTATGCCTCAGA |
| B3_Ptep_waist-less_multiplexed | ATGTCCCATTTGGGCATAAAATTGTTTCCACTCAACTTTAACCCG |
| B3_Ptep_waist-less_multiplexed | GTCCCTGCCTCTATATCTTTCTACCATTCAGAAGGCGATTGTAAT |
| B3_Ptep_waist-less_multiplexed | TGTGAACTGTCACCACAAGGTAAAATTCCACTCAACTTTAACCCG |
| B3_Ptep_waist-less_multiplexed | GTCCCTGCCTCTATATCTTTAGGAGGATGGTGACACAGAAGCTAA |
| B3_Ptep_waist-less_multiplexed | TGGGTGGTAAAACGGATGGGGATCCTTCCACTCAACTTTAACCCG |
| B3_Ptep_waist-less_multiplexed | GTCCCTGCCTCTATATCTTTGAATTTGTCACCATGTGGGAAATTG |
| B3_Ptep_waist-less_multiplexed | ACTTCGCTCGTGGGAGGTGACACTATTCCACTCAACTTTAACCCG |
| B3_Ptep_waist-less_multiplexed | GTCCCTGCCTCTATATCTTTGACGATAAAGATGATTCGGCTGAAC |
| B3_Ptep_waist-less_multiplexed | AACTCTCCTGTTCTCCAAAGGGGTGTTCCACTCAACTTTAACCCG |
| B3_Ptep_waist-less_multiplexed | GTCCCTGCCTCTATATCTTTGTGAAGGATGGTATAAGTGTTCATT |
| B3_Ptep_waist-less_multiplexed | CCTGCGGAGGAAGAGGCCTGTGGTATTCCACTCAACTTTAACCCG |
| B3_Ptep_waist-less_multiplexed | GTCCCTGCCTCTATATCTTTATCAGTTTGGTGGTCGAAGAACTTT |
| B3_Ptep_waist-less_multiplexed | ACCCAACCTTCTTAATTTTTGGTTCTTCCACTCAACTTTAACCCG |
| B3_Ptep_waist-less_multiplexed | GTCCCTGCCTCTATATCTTTGCGCTAGTGACCAGATTTTTGGTTT |
| B3_Ptep_waist-less_multiplexed | GTGGTGGGCTCTCTGAGGTGGCAGTTTCCACTCAACTTTAACCCG |
| B3_Ptep_waist-less_multiplexed | GTCCCTGCCTCTATATCTTTAAGTTCAGGGAATTTGGTTTCTGAG |
| B3_Ptep_waist-less_multiplexed | AATTGATTCTGATTTGATGTGTTCATTCCACTCAACTTTAACCCG |
| B3_Ptep_waist-less_multiplexed | GTCCCTGCCTCTATATCTTTTCGGGAGCACTGAGGCTGTCACCAA |
| B3_Ptep_waist-less_multiplexed | TCAAGCGCTTTCATTGGAGACCTGTTTCCACTCAACTTTAACCCG |
| B3_Ptep_waist-less_multiplexed | GTCCCTGCCTCTATATCTTTCATCGTCCTTCATACTGTCTCGTCT |
| B3_Ptep_waist-less_multiplexed | GGGAGGTGCATTTCTGTTGTCGGTTTTCCACTCAACTTTAACCCG |
| B3_Ptep_waist-less_multiplexed | GTCCCTGCCTCTATATCTTTTTGATTTTCATTCGAATCGCTGAGT |
| B3_Ptep_waist-less_multiplexed | TCTACTGGAGTCACTTTCCTGGCATTTCCACTCAACTTTAACCCG |
| B3_Ptep_waist-less_multiplexed | GTCCCTGCCTCTATATCTTTTCATCTTTATCATCATCTTCATCCA |
| B3_Ptep_waist-less_multiplexed | CTCTTAAGGTCATCCCCACTCTGCTTTCCACTCAACTTTAACCCG |
| B3_Ptep_waist-less_multiplexed | GTCCCTGCCTCTATATCTTTATCTCCGTACGGAGAATATGCGGCG |
| B3_Ptep_waist-less_multiplexed | TTTTTCACTATCAACAGGATCAAACTTCCACTCAACTTTAACCCG |
| B3_Ptep_waist-less_multiplexed | GTCCCTGCCTCTATATCTTTAGCCCGAGGATCTTTTATGTCATAA |
| B3_Ptep_waist-less_multiplexed | GTGTCCTGGTGGTAGGGATGCCCAGTTCCACTCAACTTTAACCCG |
| B3_Ptep_waist-less_multiplexed | GTCCCTGCCTCTATATCTTTGTGGCATGCTGTTTGAGGCTGGATA |
| B3_Ptep_waist-less_multiplexed | GATGGGCCTGACTTACTAGTGTGTCTTCCACTCAACTTTAACCCG |
| B3_Ptep_waist-less_multiplexed | GTCCCTGCCTCTATATCTTTTTGAGGAGATGCAGTATAGGCGAAA |
| B3_Ptep_waist-less_multiplexed | GCTGGGGTGCTGGCCTGTCATCAGATTCCACTCAACTTTAACCCG |
|  |  |
|  |  |
| **Initiator and gene name** | **Sequence** |
| B1_Ptep_Irx2-1_multiplexed | GAGGAGGGCAGCAAACGGAATCCCGTTGCAGAATAATCCTCAGGT |
| B1_Ptep_Irx2-1_multiplexed | TTATTGCAGGGATGTCGCCTTGAAATAGAAGAGTCTTCCTTTACG |
| B1_Ptep_Irx2-1_multiplexed | GAGGAGGGCAGCAAACGGAACACCGATAGCAGATGATTGATATAG |
| B1_Ptep_Irx2-1_multiplexed | TACTGTTTTGAATACTAGCAGATACTAGAAGAGTCTTCCTTTACG |
| B1_Ptep_Irx2-1_multiplexed | GAGGAGGGCAGCAAACGGAATGAGCCGGATACCTTCATGTTTGGT |
| B1_Ptep_Irx2-1_multiplexed | ACCACCTTGTCCACCCAGGAAGCCATAGAAGAGTCTTCCTTTACG |
| B1_Ptep_Irx2-1_multiplexed | GAGGAGGGCAGCAAACGGAAGGCACAAAACCTTGAGCAGATGAAC |
| B1_Ptep_Irx2-1_multiplexed | GTTTGAGGTGGTGTGTCCGTCTGAGTAGAAGAGTCTTCCTTTACG |
| B1_Ptep_Irx2-1_multiplexed | GAGGAGGGCAGCAAACGGAAAGGATGATAACTTCCAAAGCCACCT |
| B1_Ptep_Irx2-1_multiplexed | GGGCGATGCGAATGACCCACCAGAGTAGAAGAGTCTTCCTTTACG |
| B1_Ptep_Irx2-1_multiplexed | GAGGAGGGCAGCAAACGGAAGGATTCACCGAAAACCATCCACAGT |
| B1_Ptep_Irx2-1_multiplexed | GATGCTGAGGCATTGGTCACTTGGTTAGAAGAGTCTTCCTTTACG |
| B1_Ptep_Irx2-1_multiplexed | GAGGAGGGCAGCAAACGGAATCTCTTGAGTTGGACCACCAAGCAG |
| B1_Ptep_Irx2-1_multiplexed | CTAACATGTCCATCATACTGCCGGATAGAAGAGTCTTCCTTTACG |
| B1_Ptep_Irx2-1_multiplexed | GAGGAGGGCAGCAAACGGAACCAAAGACCATATTTTTGGTTTATT |
| B1_Ptep_Irx2-1_multiplexed | GGGGACTTTTACTCGTGGCTGTGTCTAGAAGAGTCTTCCTTTACG |
| B1_Ptep_Irx2-1_multiplexed | GAGGAGGGCAGCAAACGGAACTTCGACAGCAGAAGGCGAGCTTTT |
| B1_Ptep_Irx2-1_multiplexed | TCATGGATCGACCGGACATGTCCACTAGAAGAGTCTTCCTTTACG |
| B1_Ptep_Irx2-1_multiplexed | GAGGAGGGCAGCAAACGGAAAATTAGGCGATGAGGATGAGTCAGT |
| B1_Ptep_Irx2-1_multiplexed | TGCGCATGAGGGCCTGAATGTCCGGTAGAAGAGTCTTCCTTTACG |
| B1_Ptep_Irx2-1_multiplexed | GAGGAGGGCAGCAAACGGAAAGTACTGTCCCTAGTACTGTTTTCT |
| B1_Ptep_Irx2-1_multiplexed | TGTGAATTCGGAGGTAGCGATGAGGTAGAAGAGTCTTCCTTTACG |
| B1_Ptep_Irx2-1_multiplexed | GAGGAGGGCAGCAAACGGAATTTCTGTGGTGAGGCAGGTGGGGAT |
| B1_Ptep_Irx2-1_multiplexed | TGTTGAGCACCGTTTGGAGTTATTTTAGAAGAGTCTTCCTTTACG |
| B1_Ptep_Irx2-1_multiplexed | GAGGAGGGCAGCAAACGGAACGTAATGTACTGCACCATCAATGGA |
| B1_Ptep_Irx2-1_multiplexed | GTCGTCCAGTTATGTGATTACCTGATAGAAGAGTCTTCCTTTACG |
| B1_Ptep_Irx2-1_multiplexed | GAGGAGGGCAGCAAACGGAATTCGTCTTCGCTTTCACATTTTGTC |
| B1_Ptep_Irx2-1_multiplexed | ATCACACGATGCTGTTCTACGATCGTAGAAGAGTCTTCCTTTACG |
| B1_Ptep_Irx2-1_multiplexed | GAGGAGGGCAGCAAACGGAACTGTCCATCCTGTTCCTTTTAGTTG |
| B1_Ptep_Irx2-1_multiplexed | CTGGACTCCCCATCAACGCGATCGTTAGAAGAGTCTTCCTTTACG |
| B1_Ptep_Irx2-1_multiplexed | GAGGAGGGCAGCAAACGGAACATCGTCATAATCCTTGACGTCATT |
| B1_Ptep_Irx2-1_multiplexed | CATCACCGTCATCTTCGACGTCATCTAGAAGAGTCTTCCTTTACG |
| B1_Ptep_Irx2-1_multiplexed | GAGGAGGGCAGCAAACGGAATACCCGTAAGCAGCCAGGCTTGGAT |
| B1_Ptep_Irx2-1_multiplexed | TTTTTGCCATTGAAATCTAAACCACTAGAAGAGTCTTCCTTTACG |
| B1_Ptep_Irx2-1_multiplexed | GAGGAGGGCAGCAAACGGAACTCTCCACGGGTCGCCAGCATCTTT |
| B1_Ptep_Irx2-1_multiplexed | AATAATAAGAAGCAGATGGCGTAATTAGAAGAGTCTTCCTTTACG |
| B1_Ptep_Irx2-1_multiplexed | GAGGAGGGCAGCAAACGGAAAAAGGCTGAAGATTCAACTCCCGTG |
| B1_Ptep_Irx2-1_multiplexed | ACCATAGGCTGTACTCAAAGAGGGATAGAAGAGTCTTCCTTTACG |
| B1_Ptep_Irx2-1_multiplexed | GAGGAGGGCAGCAAACGGAACTAGGATGGTAGGTTGTAAGTAGCC |
| B1_Ptep_Irx2-1_multiplexed | TTGGGATAGGCTTGATGCTCGTAGATAGAAGAGTCTTCCTTTACG |
|  |  |
| B2_Ptep_Irx3-1_multiplexed | CCTCGTAAATCCTCATCAAATAACGAGGCGGAATGTTCGAGAACC |
| B2_Ptep_Irx3-1_multiplexed | TTATTCACTATGTTTAACATTCGTTAAATCATCCAGTAAACCGCC |
| B2_Ptep_Irx3-1_multiplexed | CCTCGTAAATCCTCATCAAAAATCCGTTCGAATCGAAAAATTCCG |
| B2_Ptep_Irx3-1_multiplexed | TGACAGCACTTTTGAAGTGTTCTATAAATCATCCAGTAAACCGCC |
| B2_Ptep_Irx3-1_multiplexed | CCTCGTAAATCCTCATCAAATGATTTGTTGTCATACTCTAAACAG |
| B2_Ptep_Irx3-1_multiplexed | TGTGTCACAATCCGCTTCGTAAACTAAATCATCCAGTAAACCGCC |
| B2_Ptep_Irx3-1_multiplexed | CCTCGTAAATCCTCATCAAATTCTGAGGCACACTTTATATCATTC |
| B2_Ptep_Irx3-1_multiplexed | GGGGTTGTCCATCATGCACGATCCTAAATCATCCAGTAAACCGCC |
| B2_Ptep_Irx3-1_multiplexed | CCTCGTAAATCCTCATCAAAGGCATGAAATCTGTAAAAGAGGACA |
| B2_Ptep_Irx3-1_multiplexed | TTTTTATTGCAACACAACTCACTGTAAATCATCCAGTAAACCGCC |
| B2_Ptep_Irx3-1_multiplexed | CCTCGTAAATCCTCATCAAAGGACATTTTCTAAACAGAAATCCAT |
| B2_Ptep_Irx3-1_multiplexed | TCGAGTTCACGGAACACTCTGCTTTAAATCATCCAGTAAACCGCC |
| B2_Ptep_Irx3-1_multiplexed | CCTCGTAAATCCTCATCAAACAAGCGACCAAATTTTTGGTCTGCT |
| B2_Ptep_Irx3-1_multiplexed | GGGGACTATTGGATGTGGCAGTTCTAAATCATCCAGTAAACCGCC |
| B2_Ptep_Irx3-1_multiplexed | CCTCGTAAATCCTCATCAAAGGAAACGTCTTTTGCACTAGGTTTT |
| B2_Ptep_Irx3-1_multiplexed | GGAATCGAAGGTTTCCGAAAACCTAAAATCATCCAGTAAACCGCC |
| B2_Ptep_Irx3-1_multiplexed | CCTCGTAAATCCTCATCAAATCCTAACTGGGCTAGAATGTTCTGA |
| B2_Ptep_Irx3-1_multiplexed | CCTGCTGGAATCCCGGGTAGCAGGAAAATCATCCAGTAAACCGCC |
| B2_Ptep_Irx3-1_multiplexed | CCTCGTAAATCCTCATCAAAAGTTCGATAACATTCCAGAGCACAT |
| B2_Ptep_Irx3-1_multiplexed | GATGGAATCTTCTGATGAAAGACTTAAATCATCCAGTAAACCGCC |
| B2_Ptep_Irx3-1_multiplexed | CCTCGTAAATCCTCATCAAAAAGGTTCTTTGCTGTGGAATGTTTC |
| B2_Ptep_Irx3-1_multiplexed | GGGGAGATGTGGAATAATCGGCAGGAAATCATCCAGTAAACCGCC |
| B2_Ptep_Irx3-1_multiplexed | CCTCGTAAATCCTCATCAAACTTCTGCTGTATCGTCTGACTTCAG |
| B2_Ptep_Irx3-1_multiplexed | AAAAATCGTTCAAGTTGCTGCACTCAAATCATCCAGTAAACCGCC |
| B2_Ptep_Irx3-1_multiplexed | CCTCGTAAATCCTCATCAAACTGTCTGTATAGGTTGTACAGGAGG |
| B2_Ptep_Irx3-1_multiplexed | TTTTTAACACTCTCCATAGAACCGTAAATCATCCAGTAAACCGCC |
| B2_Ptep_Irx3-1_multiplexed | CCTCGTAAATCCTCATCAAATCCATGAGTTTCGTGATTCTTTCAA |
| B2_Ptep_Irx3-1_multiplexed | TAGGATCGTATGGGTAACAGGATGCAAATCATCCAGTAAACCGCC |
| B2_Ptep_Irx3-1_multiplexed | CCTCGTAAATCCTCATCAAAAGTGGTTGTGTTGGCTGCCAGTGAT |
| B2_Ptep_Irx3-1_multiplexed | GTAAGGAGCACCCAGTGTTGAGTAGAAATCATCCAGTAAACCGCC |
| B2_Ptep_Irx3-1_multiplexed | CCTCGTAAATCCTCATCAAAAGACCGAAGGATGCCAGCCGAGGTA |
| B2_Ptep_Irx3-1_multiplexed | ATGTGATCAGCGTATGAAGAACCGTAAATCATCCAGTAAACCGCC |
| B2_Ptep_Irx3-1_multiplexed | CCTCGTAAATCCTCATCAAATACCTGTGTTGAAGAAGGTGGATAT |
| B2_Ptep_Irx3-1_multiplexed | GCAACACGGTCCGCCGCTGCTCACCAAATCATCCAGTAAACCGCC |
| B2_Ptep_Irx3-1_multiplexed | CCTCGTAAATCCTCATCAAACGATTTGGACGGATTAGTGGGGTGT |
| B2_Ptep_Irx3-1_multiplexed | TAACCGAATTGTGAATAGGACATTAAAATCATCCAGTAAACCGCC |
| B2_Ptep_Irx3-1_multiplexed | CCTCGTAAATCCTCATCAAATCATTCGGCCTTTCTTGAACCGATC |
| B2_Ptep_Irx3-1_multiplexed | GAAAGGGAGATCGGCCTCACAGTGTAAATCATCCAGTAAACCGCC |
| B2_Ptep_Irx3-1_multiplexed | CCTCGTAAATCCTCATCAAAATTCCGTTATTCCAACTGTATCCCT |
| B2_Ptep_Irx3-1_multiplexed | CAAAAACATGGCGATCATCGGTCAAAAATCATCCAGTAAACCGCC |
|  |  |
| B3_Ptep_waist-less_multiplexed | GTCCCTGCCTCTATATCTTTATCTTCTAATTTCGTCCTATTGGGT |
| B3_Ptep_waist-less_multiplexed | TTAGCTCTCACACCGACTTGGAATATTCCACTCAACTTTAACCCG |
| B3_Ptep_waist-less_multiplexed | GTCCCTGCCTCTATATCTTTGCTGGAGCACTTGTAAAACCAACGG |
| B3_Ptep_waist-less_multiplexed | GATGTAGAAACTGTATGATACATAGTTCCACTCAACTTTAACCCG |
| B3_Ptep_waist-less_multiplexed | GTCCCTGCCTCTATATCTTTCTGGCTGAAATGTCCTTATGTCTAG |
| B3_Ptep_waist-less_multiplexed | ATGAAAAAGAGGATGTCCTCAAGAATTCCACTCAACTTTAACCCG |
| B3_Ptep_waist-less_multiplexed | GTCCCTGCCTCTATATCTTTATAGTCTATAGGATTATGCCTCAGA |
| B3_Ptep_waist-less_multiplexed | ATGTCCCATTTGGGCATAAAATTGTTTCCACTCAACTTTAACCCG |
| B3_Ptep_waist-less_multiplexed | GTCCCTGCCTCTATATCTTTCTACCATTCAGAAGGCGATTGTAAT |
| B3_Ptep_waist-less_multiplexed | TGTGAACTGTCACCACAAGGTAAAATTCCACTCAACTTTAACCCG |
| B3_Ptep_waist-less_multiplexed | GTCCCTGCCTCTATATCTTTAGGAGGATGGTGACACAGAAGCTAA |
| B3_Ptep_waist-less_multiplexed | TGGGTGGTAAAACGGATGGGGATCCTTCCACTCAACTTTAACCCG |
| B3_Ptep_waist-less_multiplexed | GTCCCTGCCTCTATATCTTTGAATTTGTCACCATGTGGGAAATTG |
| B3_Ptep_waist-less_multiplexed | ACTTCGCTCGTGGGAGGTGACACTATTCCACTCAACTTTAACCCG |
| B3_Ptep_waist-less_multiplexed | GTCCCTGCCTCTATATCTTTGACGATAAAGATGATTCGGCTGAAC |
| B3_Ptep_waist-less_multiplexed | AACTCTCCTGTTCTCCAAAGGGGTGTTCCACTCAACTTTAACCCG |
| B3_Ptep_waist-less_multiplexed | GTCCCTGCCTCTATATCTTTGTGAAGGATGGTATAAGTGTTCATT |
| B3_Ptep_waist-less_multiplexed | CCTGCGGAGGAAGAGGCCTGTGGTATTCCACTCAACTTTAACCCG |
| B3_Ptep_waist-less_multiplexed | GTCCCTGCCTCTATATCTTTATCAGTTTGGTGGTCGAAGAACTTT |
| B3_Ptep_waist-less_multiplexed | ACCCAACCTTCTTAATTTTTGGTTCTTCCACTCAACTTTAACCCG |
| B3_Ptep_waist-less_multiplexed | GTCCCTGCCTCTATATCTTTGCGCTAGTGACCAGATTTTTGGTTT |
| B3_Ptep_waist-less_multiplexed | GTGGTGGGCTCTCTGAGGTGGCAGTTTCCACTCAACTTTAACCCG |
| B3_Ptep_waist-less_multiplexed | GTCCCTGCCTCTATATCTTTAAGTTCAGGGAATTTGGTTTCTGAG |
| B3_Ptep_waist-less_multiplexed | AATTGATTCTGATTTGATGTGTTCATTCCACTCAACTTTAACCCG |
| B3_Ptep_waist-less_multiplexed | GTCCCTGCCTCTATATCTTTTCGGGAGCACTGAGGCTGTCACCAA |
| B3_Ptep_waist-less_multiplexed | TCAAGCGCTTTCATTGGAGACCTGTTTCCACTCAACTTTAACCCG |
| B3_Ptep_waist-less_multiplexed | GTCCCTGCCTCTATATCTTTCATCGTCCTTCATACTGTCTCGTCT |
| B3_Ptep_waist-less_multiplexed | GGGAGGTGCATTTCTGTTGTCGGTTTTCCACTCAACTTTAACCCG |
| B3_Ptep_waist-less_multiplexed | GTCCCTGCCTCTATATCTTTTTGATTTTCATTCGAATCGCTGAGT |
| B3_Ptep_waist-less_multiplexed | TCTACTGGAGTCACTTTCCTGGCATTTCCACTCAACTTTAACCCG |
| B3_Ptep_waist-less_multiplexed | GTCCCTGCCTCTATATCTTTTCATCTTTATCATCATCTTCATCCA |
| B3_Ptep_waist-less_multiplexed | CTCTTAAGGTCATCCCCACTCTGCTTTCCACTCAACTTTAACCCG |
| B3_Ptep_waist-less_multiplexed | GTCCCTGCCTCTATATCTTTATCTCCGTACGGAGAATATGCGGCG |
| B3_Ptep_waist-less_multiplexed | TTTTTCACTATCAACAGGATCAAACTTCCACTCAACTTTAACCCG |
| B3_Ptep_waist-less_multiplexed | GTCCCTGCCTCTATATCTTTAGCCCGAGGATCTTTTATGTCATAA |
| B3_Ptep_waist-less_multiplexed | GTGTCCTGGTGGTAGGGATGCCCAGTTCCACTCAACTTTAACCCG |
| B3_Ptep_waist-less_multiplexed | GTCCCTGCCTCTATATCTTTGTGGCATGCTGTTTGAGGCTGGATA |
| B3_Ptep_waist-less_multiplexed | GATGGGCCTGACTTACTAGTGTGTCTTCCACTCAACTTTAACCCG |
| B3_Ptep_waist-less_multiplexed | GTCCCTGCCTCTATATCTTTTTGAGGAGATGCAGTATAGGCGAAA |
| B3_Ptep_waist-less_multiplexed | GCTGGGGTGCTGGCCTGTCATCAGATTCCACTCAACTTTAACCCG |
|  |  |
|  |  |
| **Initiator and gene name** | **Sequence** |
| B2_Popi_irx2_multiplexed | CCTCGTAAATCCTCATCAAATCTTCGGAGAGGGAAAGCCTCTGAT |
| B2_Popi_irx2_multiplexed | GAAGCGCACGGAGACGAAGATTCGTAAATCATCCAGTAAACCGCC |
| B2_Popi_irx2_multiplexed | CCTCGTAAATCCTCATCAAATCTAACGTTCTTGACATCGTGAAGG |
| B2_Popi_irx2_multiplexed | CCCCGGTACTTCGCACATCATCGATAAATCATCCAGTAAACCGCC |
| B2_Popi_irx2_multiplexed | CCTCGTAAATCCTCATCAAAGTAATTGTTCAACTGATGTCCGACT |
| B2_Popi_irx2_multiplexed | ATGGTCACTCGGAGCCCCTACACCAAAATCATCCAGTAAACCGCC |
| B2_Popi_irx2_multiplexed | CCTCGTAAATCCTCATCAAACAATGTTTCTGATACGGTTGGTTCG |
| B2_Popi_irx2_multiplexed | GGGTAGTTGAAAGTTTCTCTGTTCAAAATCATCCAGTAAACCGCC |
| B2_Popi_irx2_multiplexed | CCTCGTAAATCCTCATCAAAAGCGGCTAAAGACCATATTTTTGGC |
| B2_Popi_irx2_multiplexed | GGGTGGAGGACTCTTACTGGTAGCCAAATCATCCAGTAAACCGCC |
| B2_Popi_irx2_multiplexed | CCTCGTAAATCCTCATCAAAACGCGTCTTTAATGAAAGCGTCCAA |
| B2_Popi_irx2_multiplexed | TCTCGCCCGACGAGGTGGATGACAGAAATCATCCAGTAAACCGCC |
| B2_Popi_irx2_multiplexed | CCTCGTAAATCCTCATCAAATTTGTTTGTTCCGATGATACTTGGT |
| B2_Popi_irx2_multiplexed | GTAAAGGCCGAGGTGCCCGTTAAAAAAATCATCCAGTAAACCGCC |
| B2_Popi_irx2_multiplexed | CCTCGTAAATCCTCATCAAAGATGAAGCGGAAGGTACCGATGGTA |
| B2_Popi_irx2_multiplexed | GTCGAATGATGAGGCCGAAATGGTCAAATCATCCAGTAAACCGCC |
| B2_Popi_irx2_multiplexed | CCTCGTAAATCCTCATCAAACAAACGAGATCCATTTTGAGACTCG |
| B2_Popi_irx2_multiplexed | ATAGCCGCTGTGCAATCCCAACGTTAAATCATCCAGTAAACCGCC |
| B2_Popi_irx2_multiplexed | CCTCGTAAATCCTCATCAAATTCGATCATCGTATTCGTCATTCCT |
| B2_Popi_irx2_multiplexed | GTGTCCTGCCAAGACACGCTAACCTAAATCATCCAGTAAACCGCC |
| B2_Popi_irx2_multiplexed | CCTCGTAAATCCTCATCAAAAAAATCACCTCCTTGGTCTTTCGCT |
| B2_Popi_irx2_multiplexed | TTGTCCAATCTTTCTTGCTTTGTTGAAATCATCCAGTAAACCGCC |
| B2_Popi_irx2_multiplexed | CCTCGTAAATCCTCATCAAACAGTTTGACTATCATCTCCGTGGTC |
| B2_Popi_irx2_multiplexed | CATCTTCGGATCTTCTACTACCAGAAAATCATCCAGTAAACCGCC |
| B2_Popi_irx2_multiplexed | CCTCGTAAATCCTCATCAAAAACCTCCGTACCCATACGCTGCCAA |
| B2_Popi_irx2_multiplexed | TTTTTCGTCTTGCCCCATTAAAATCAAATCATCCAGTAAACCGCC |
| B2_Popi_irx2_multiplexed | CCTCGTAAATCCTCATCAAATTGATGAGCATTCGCCGGATGAGCA |
| B2_Popi_irx2_multiplexed | TGGGTCGTAGTAATACGAAGCTGTAAAATCATCCAGTAAACCGCC |
| B2_Popi_irx2_multiplexed | CCTCGTAAATCCTCATCAAATCTTTCATTCCAAATCCAGTACTCA |
| B2_Popi_irx2_multiplexed | GAAGACAACGTTCCCCTCCACGGATAAATCATCCAGTAAACCGCC |
| B2_Popi_irx2_multiplexed | CCTCGTAAATCCTCATCAAATAGCCAAACTAGCGTATCCGTTACT |
| B2_Popi_irx2_multiplexed | TTGGATAAAAGGCGGAAGAATCAACAAATCATCCAGTAAACCGCC |
| B2_Popi_irx2_multiplexed | CCTCGTAAATCCTCATCAAAGTGGATGAACTTGATGCGAGTGATT |
| B2_Popi_irx2_multiplexed | TCCCGTAGAATCCGGCGGCCGTCAAAAATCATCCAGTAAACCGCC |
| B2_Popi_irx2_multiplexed | CCTCGTAAATCCTCATCAAACGCCGTGGATATCAACCTGGATTCG |
| B2_Popi_irx2_multiplexed | GGAATGGATAGAAGGGTGGTGATACAAATCATCCAGTAAACCGCC |
| B2_Popi_irx2_multiplexed | CCTCGTAAATCCTCATCAAAGGCGATTGGGGTGAAGGTGAAGAAA |
| B2_Popi_irx2_multiplexed | GAAGACACGGTGGCTTCAACGCAAGAAATCATCCAGTAAACCGCC |
| B2_Popi_irx2_multiplexed | CCTCGTAAATCCTCATCAAAACCACCGGTTGATACGGTCAACATT |
| B2_Popi_irx2_multiplexed | CGTGCAACTGGCACCACCAGTAACAAAATCATCCAGTAAACCGCC |
|  |  |
| B3_popi_irx3_multiplexed | GTCCCTGCCTCTATATCTTTATCAGTTCTTAGATGGTCCAAGGCG |
| B3_popi_irx3_multiplexed | TTATTGGACTTGAGCCGAACCGTCGTTCCACTCAACTTTAACCCG |
| B3_popi_irx3_multiplexed | GTCCCTGCCTCTATATCTTTGTCGATCGTGAGTTTGAGATTCGCG |
| B3_popi_irx3_multiplexed | GTGATGTTCTGACCAAAGGAGGCGGTTCCACTCAACTTTAACCCG |
| B3_popi_irx3_multiplexed | GTCCCTGCCTCTATATCTTTCATGCCATGAGGCGAGGTAATATCA |
| B3_popi_irx3_multiplexed | AAGATCGTAAGCCATGGCTGACGAGTTCCACTCAACTTTAACCCG |
| B3_popi_irx3_multiplexed | GTCCCTGCCTCTATATCTTTTGCGACACTCGACGATGAAATAATG |
| B3_popi_irx3_multiplexed | TAAGCCCTAGACGCCTCATTTCCCTTTCCACTCAACTTTAACCCG |
| B3_popi_irx3_multiplexed | GTCCCTGCCTCTATATCTTTCGGTCGTTGCTGGATCGAAAGCCGA |
| B3_popi_irx3_multiplexed | ATTGTTCCTTGGATTCGTCGTCGCCTTCCACTCAACTTTAACCCG |
| B3_popi_irx3_multiplexed | GTCCCTGCCTCTATATCTTTACCGCGTGTCCCATCGTTCCGTTCA |
| B3_popi_irx3_multiplexed | ACTGGTGACGTCGACGAACAGTTTTTTCCACTCAACTTTAACCCG |
| B3_popi_irx3_multiplexed | GTCCCTGCCTCTATATCTTTTGGCGTAATGGTCTTCGTAGTGTCC |
| B3_popi_irx3_multiplexed | AGTTTTTAGCACTCGTCACTCCGTTTTCCACTCAACTTTAACCCG |
| B3_popi_irx3_multiplexed | GTCCCTGCCTCTATATCTTTCATATCGCCGTCCATTTCCGACTTA |
| B3_popi_irx3_multiplexed | GTTGGCCACGTTGGCTCGTCCGCAATTCCACTCAACTTTAACCCG |
| B3_popi_irx3_multiplexed | GTCCCTGCCTCTATATCTTTTTGATACCGCTCGGTGTCGCGGGTA |
| B3_popi_irx3_multiplexed | TATTTAGGATCTCGATATTCCTGTCTTCCACTCAACTTTAACCCG |
| B3_popi_irx3_multiplexed | GTCCCTGCCTCTATATCTTTGCTAAGGACCATATTTTCGGTCTAT |
| B3_popi_irx3_multiplexed | GGCGGACTGTCCGAAGTGGCCGTGTTTCCACTCAACTTTAACCCG |
| B3_popi_irx3_multiplexed | GTCCCTGCCTCTATATCTTTGCAAACCACCTCTTAACAAGAGATT |
| B3_popi_irx3_multiplexed | ACGGAGGGGTGACGTGGTGACCGTGTTCCACTCAACTTTAACCCG |
| B3_popi_irx3_multiplexed | GTCCCTGCCTCTATATCTTTCAAGGGTGTCGACAACGGGTTGACG |
| B3_popi_irx3_multiplexed | TTGTTGCAAATGAAGGTTATGCGAATTCCACTCAACTTTAACCCG |
| B3_popi_irx3_multiplexed | GTCCCTGCCTCTATATCTTTCTACTCGTTGTGCCAATTCCCCTGT |
| B3_popi_irx3_multiplexed | TCCGCCGAACTTGAAGAGTTCTCCGTTCCACTCAACTTTAACCCG |
| B3_popi_irx3_multiplexed | GTCCCTGCCTCTATATCTTTAATCTCTCGACAACTCGTCTTCTTG |
| B3_popi_irx3_multiplexed | GATGATGGTGCATCTTGTAGTCGGATTCCACTCAACTTTAACCCG |
| B3_popi_irx3_multiplexed | GTCCCTGCCTCTATATCTTTGCCCGATCGCTCATCGTGAATGGAG |
| B3_popi_irx3_multiplexed | CATATGAGAACCCGTGTTCATTTTGTTCCACTCAACTTTAACCCG |
| B3_popi_irx3_multiplexed | GTCCCTGCCTCTATATCTTTACCGTCGACGCCGTCATTCGTCTTT |
| B3_popi_irx3_multiplexed | TTCATCGCCGCTGTCCGATTTGTCATTCCACTCAACTTTAACCCG |
| B3_popi_irx3_multiplexed | GTCCCTGCCTCTATATCTTTTTTCAAATCGTAGGGACTTGGCAAT |
| B3_popi_irx3_multiplexed | CAGGGATCCCCACGCTCCTCTACCGTTCCACTCAACTTTAACCCG |
| B3_popi_irx3_multiplexed | GTCCCTGCCTCTATATCTTTAGAGCGGCTGTTACGTAACTGGTTT |
| B3_popi_irx3_multiplexed | GAATAGAAGGCGGAGGCGTTGGTTCTTCCACTCAACTTTAACCCG |
| B3_popi_irx3_multiplexed | GTCCCTGCCTCTATATCTTTCTTCCACTGGTCGGGGTACTGTTAT |
| B3_popi_irx3_multiplexed | TCCGGAGACATGACACCGCTAGTAATTCCACTCAACTTTAACCCG |
| B3_popi_irx3_multiplexed | GTCCCTGCCTCTATATCTTTGTCCGGTCATAAGAAGCTGAGAGGA |
| B3_popi_irx3_multiplexed | AGCAAGTTGTGGCAACTGGAGGTTGTTCCACTCAACTTTAACCCG |
