# Supplementary figures and images for: A taxon-restricted duplicate of Iroquois3 is required for patterning the spider waist
Source: PLoS Biol. 2024 Aug 29;22(8):e3002771. doi: 10.1371/journal.pbio.3002771 (PMC11361693; doi:10.1371/journal.pbio.3002771)

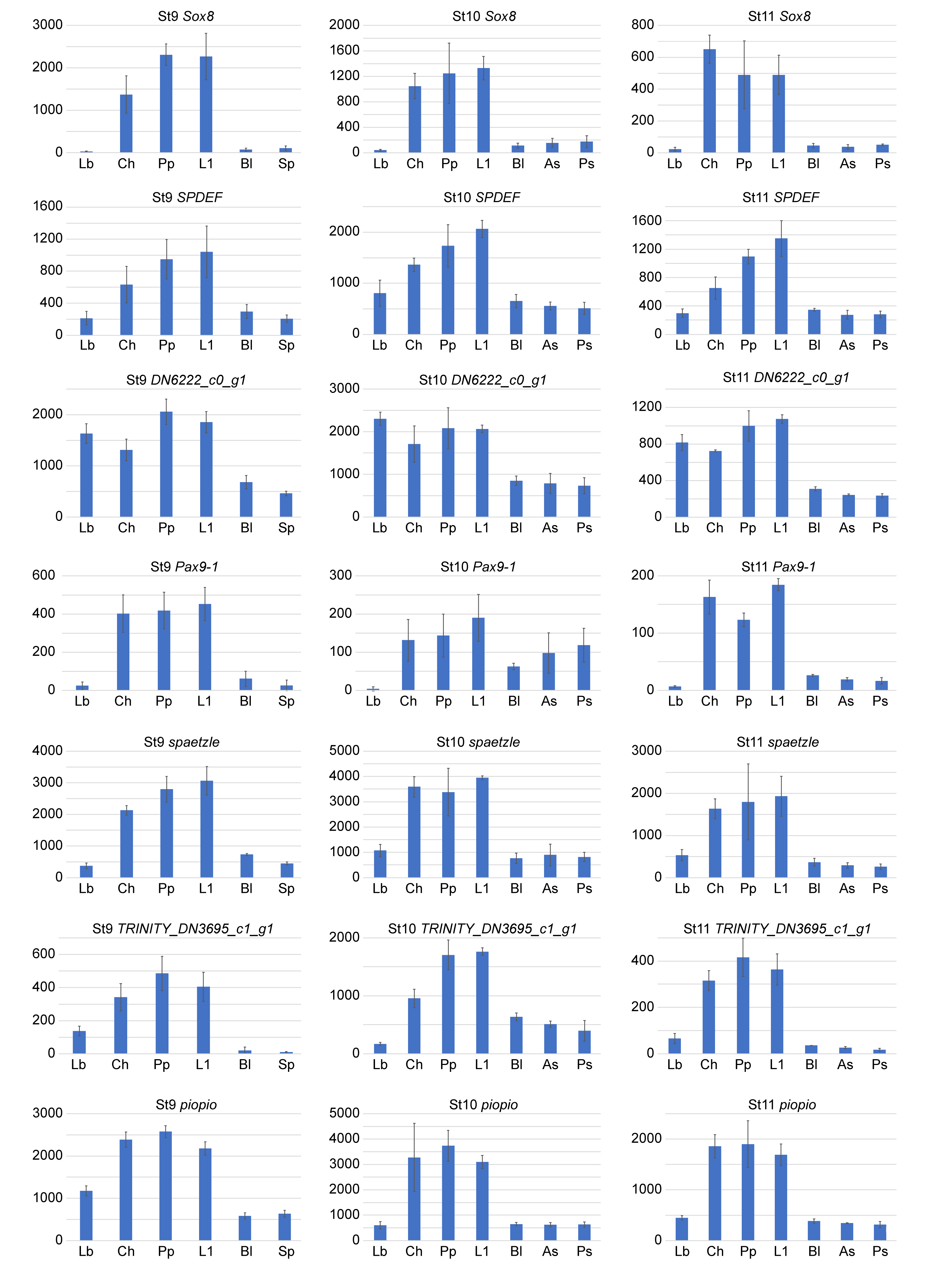

Supplement: S2 Fig — For orthologous gene identifications of uncharacterized transcripts between A. hentzi and P. tepidariorum, see S2 Table. Complete dataset is provided in S2–S4 Data. The data underlying the graphs shown in the figure can be found in S1 Data. DGE, differential gene expression; RNAi, RNA interference; TPM, transcripts per million. (TIF) [file pbio.3002771.s011.tif]

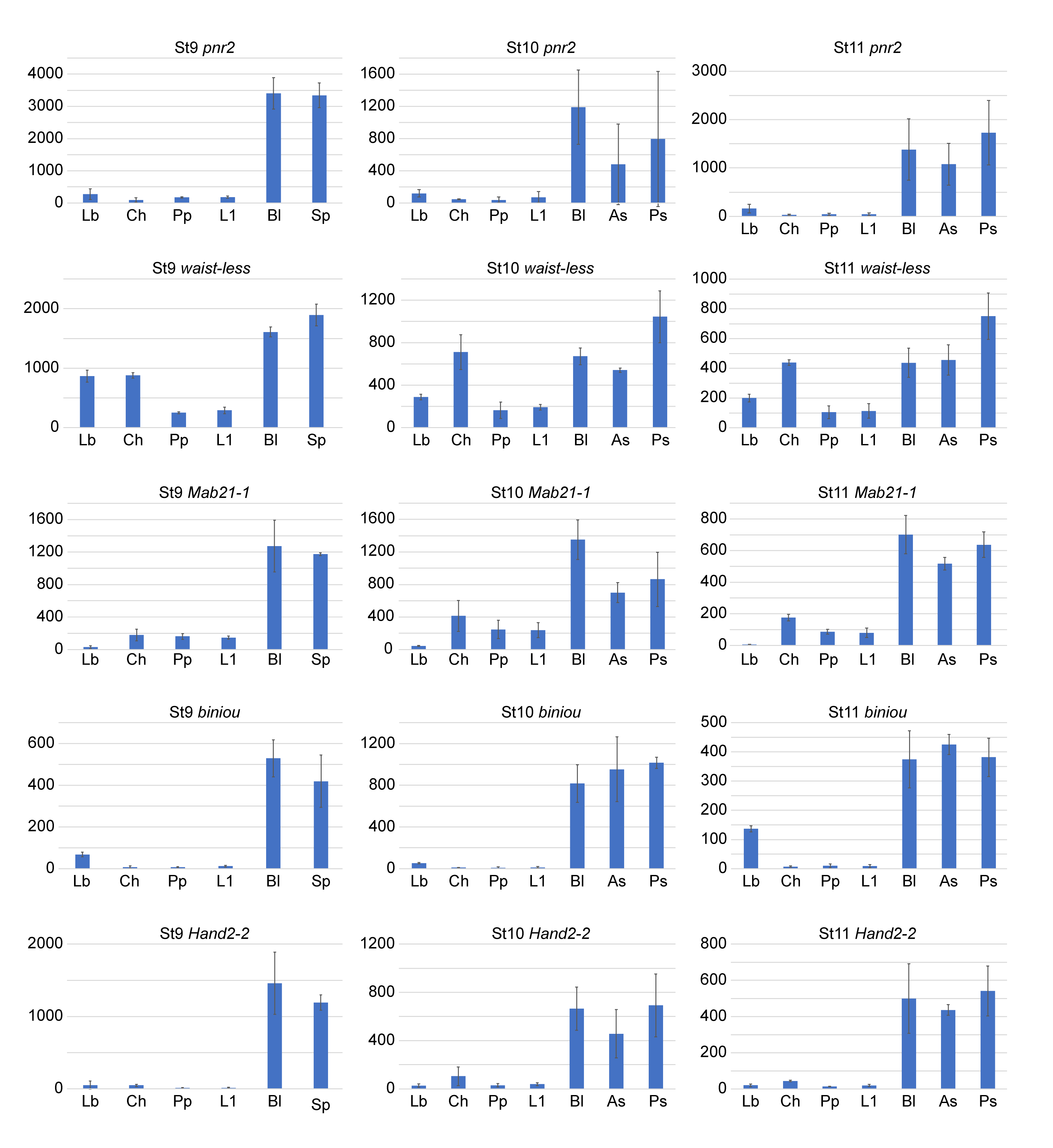

Supplement: S3 Fig — Expression levels are shown by RNA-seq library (tissue type) in TPM. RNAi screens against opisthosoma-enriched genes in P. tepidariorum resulted in 2 phenotypes (pnr2, waist-less). The remaining candidates resulted in high mortality (Mab21-1) and/or no discernable phenotype (Mab21-1, biniou, Hand2-2). For orthologous gene identifications of uncharacterized transcripts between A. hentzi and P. tepidariorum, see S2 Table. Complete dataset is provided in S2 and S3 Data. The data underlying the graphs shown in the figure can be found in S1 Data. DGE, differential gene expression; RNAi, RNA interference; TPM, transcripts per million. (TIF) [file pbio.3002771.s012.tif]

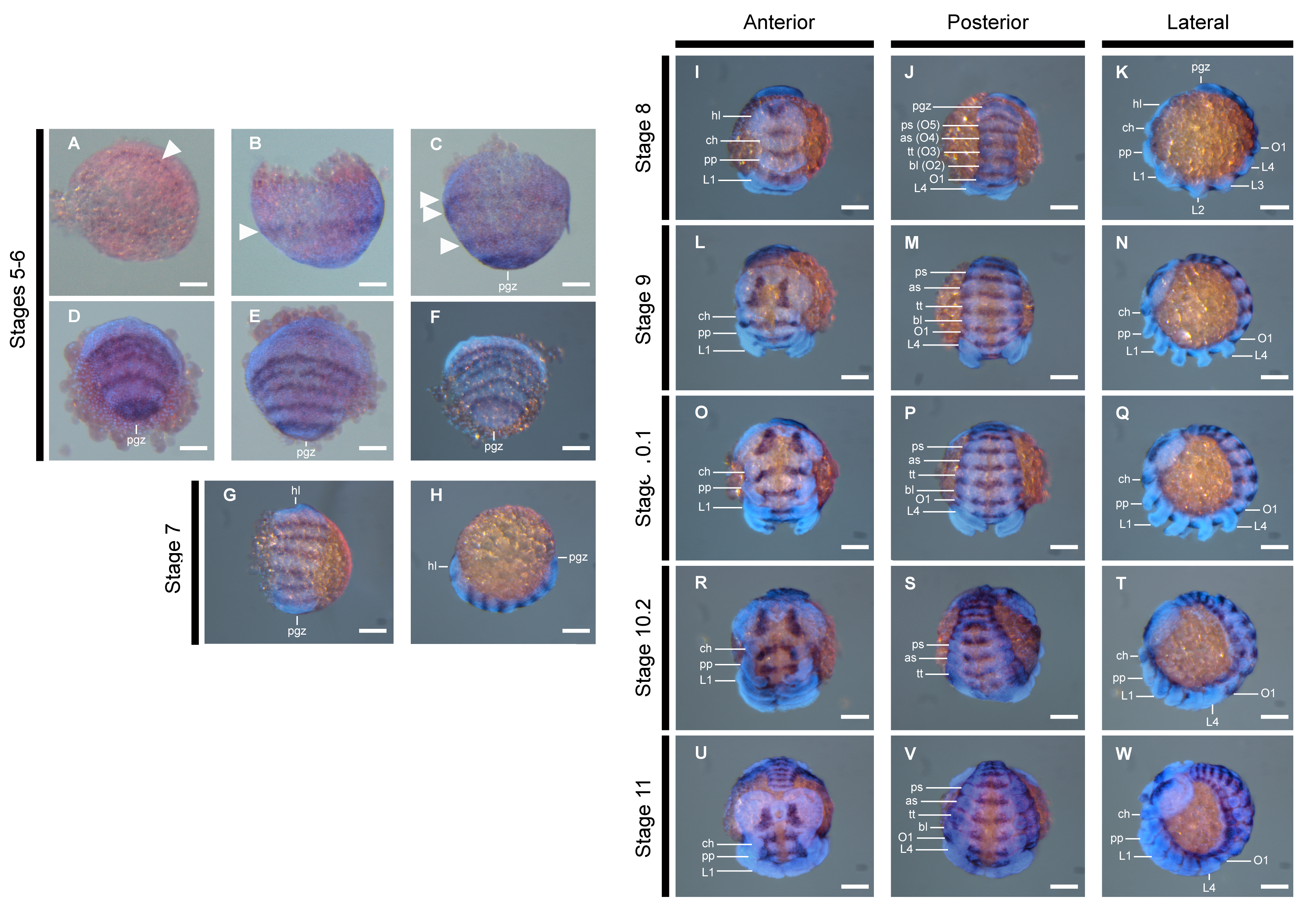

Supplement: S5 Fig — Panel A constitutes a brightfield-only image of DIG-labeled in situ hybridization for Ptep-waist-less; panels B-W constitute merged images of Hoechst and DIG-labeled in situ hybridization for Ptep-waist-less. Abbreviations as in Fig 2. Scale bar: 100 μm. (TIF) [file pbio.3002771.s014.tif]

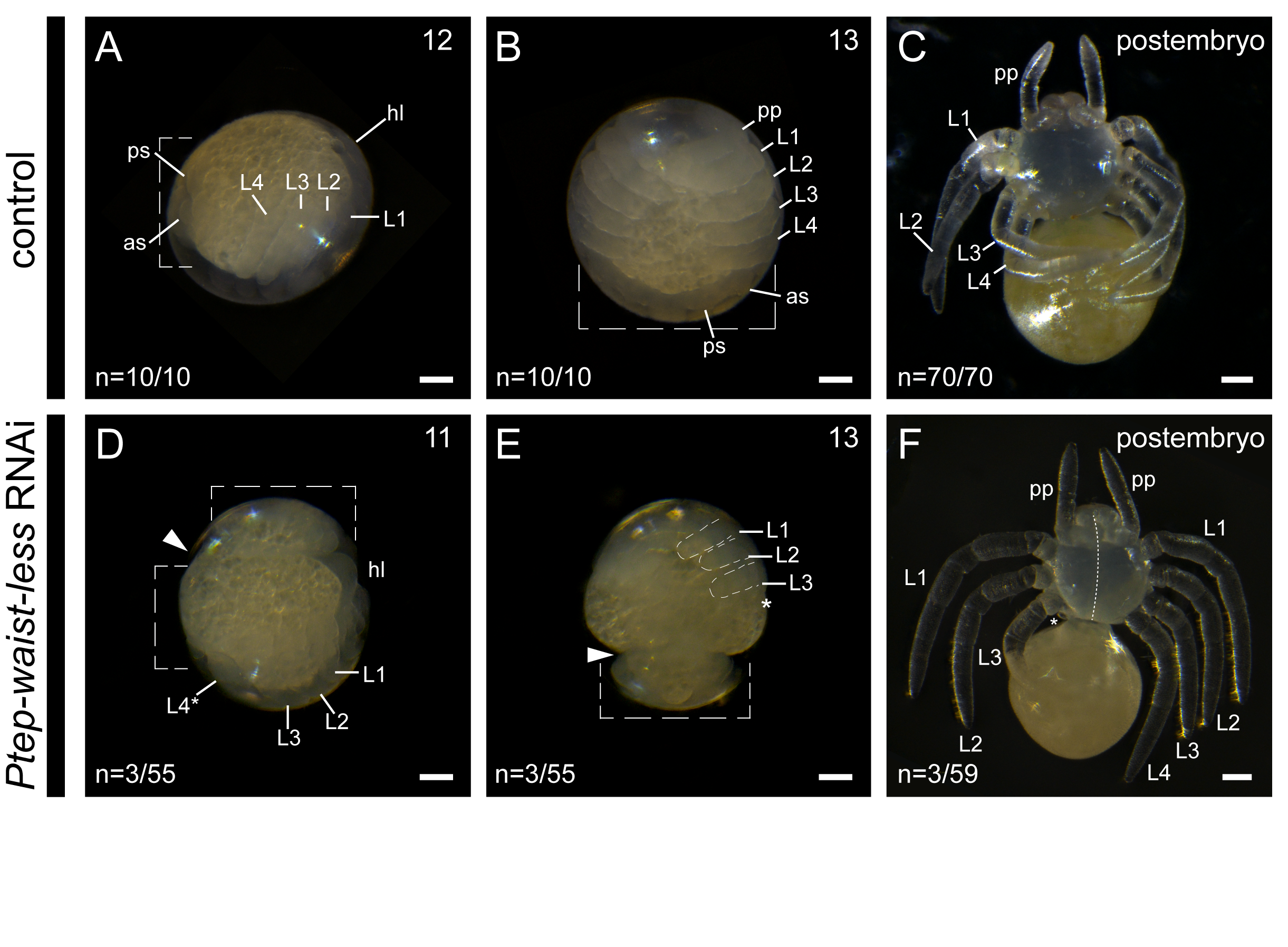

Supplement: S7 Fig — (A-C) Negative control embryos exhibiting wild-type development. (D) Stage 11 Class I RNAi embryo exhibiting discontinuous germ band and aberrant disposition of opisthosoma. (E) Stage 13 Class II RNAi embryo exhibiting anomalous development of the pedicel territory and constriction of germ band due to missing tissue between tagmata. (F) Postembryo from RNAi experiment with mosaic phenotype, exhibiting loss of L4 (asterisk) and smaller prosoma on affected side (note position of dotted line in midline of the prosoma). Scale bar: 100 μm. (TIF) [file pbio.3002771.s016.tif]

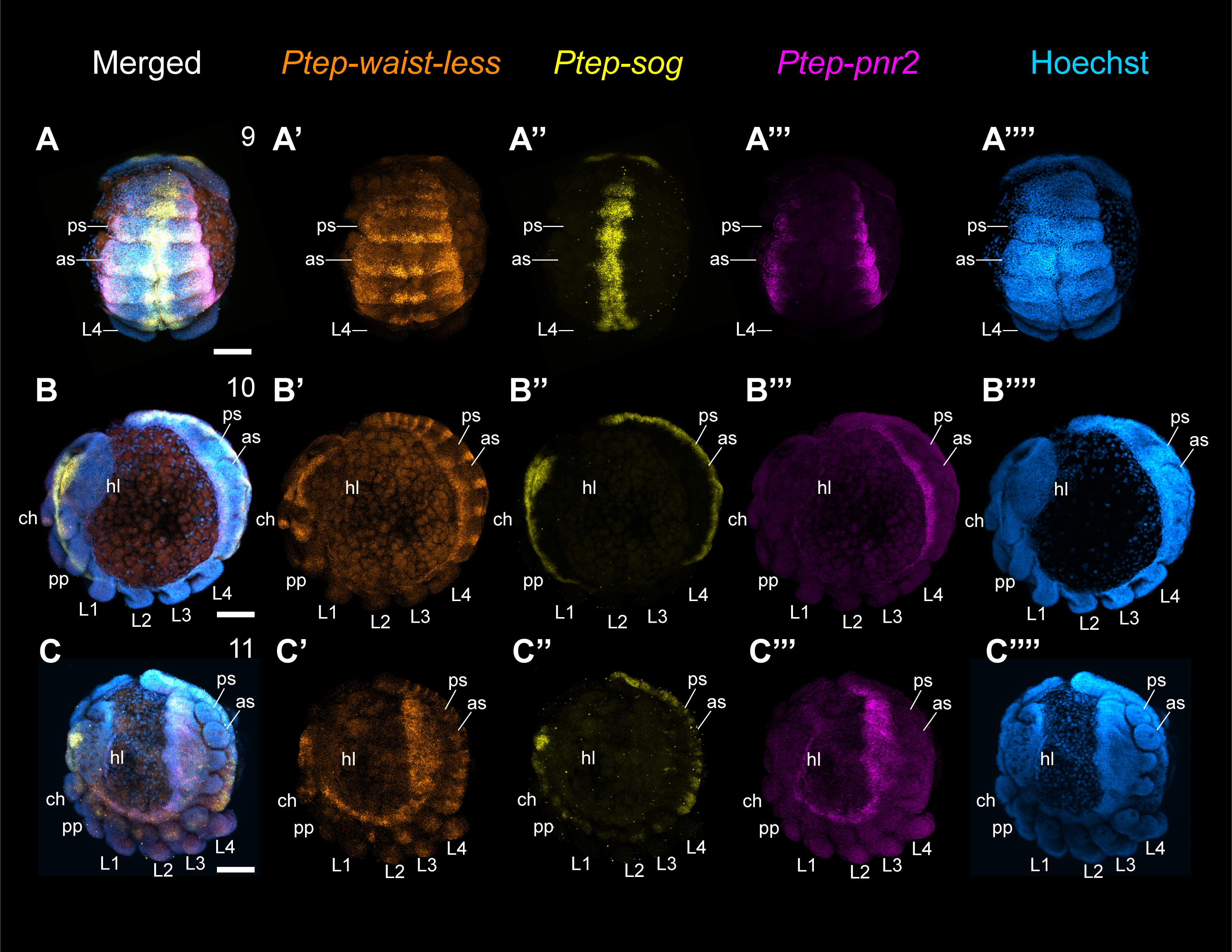

Supplement: S8 Fig — Across all 3 stages surveyed for DGE in A. hentzi, Ptep-pnr2 is expressed in the lateral edge of the germ band, which will become the dorsal part of the spider. In accordance with the DGE data, the strongest expression of Ptep-pnr2 is in the opisthosoma (n = 12/12). Abbreviations as in Fig 2. Scale bars: 100 μm. (TIF) [file pbio.3002771.s017.tif]

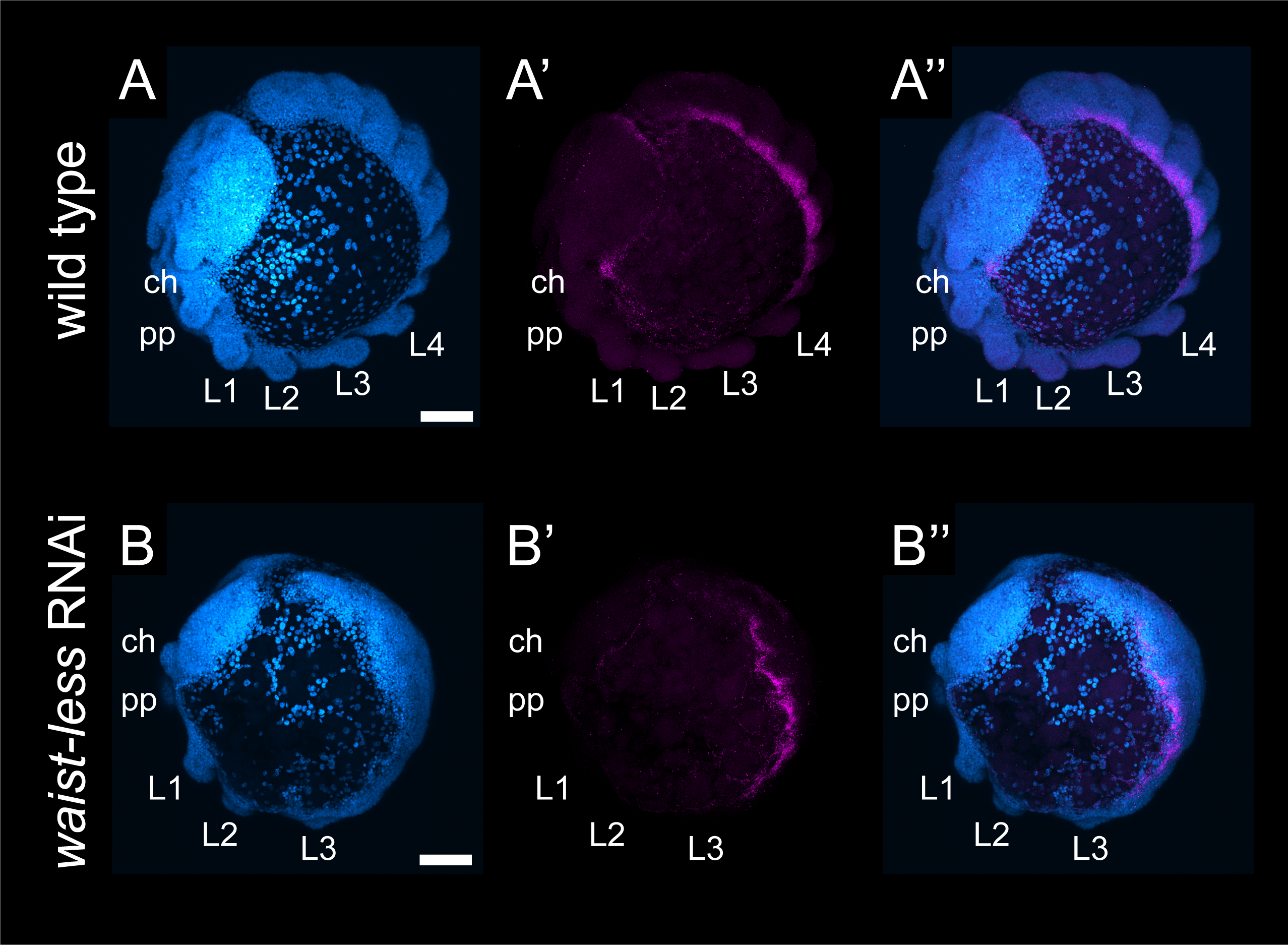

Supplement: S9 Fig — In Ptep-waist-less RNAi embryos, the expression of Ptep-pnr2 is no longer cleanly defined in the lateral edge of the opisthosoma and becomes blurred (n = 6/9; wild type n = 12/12). Abbreviations as in Fig 2. Scale bars: 100 μm. (TIF) [file pbio.3002771.s018.tif]

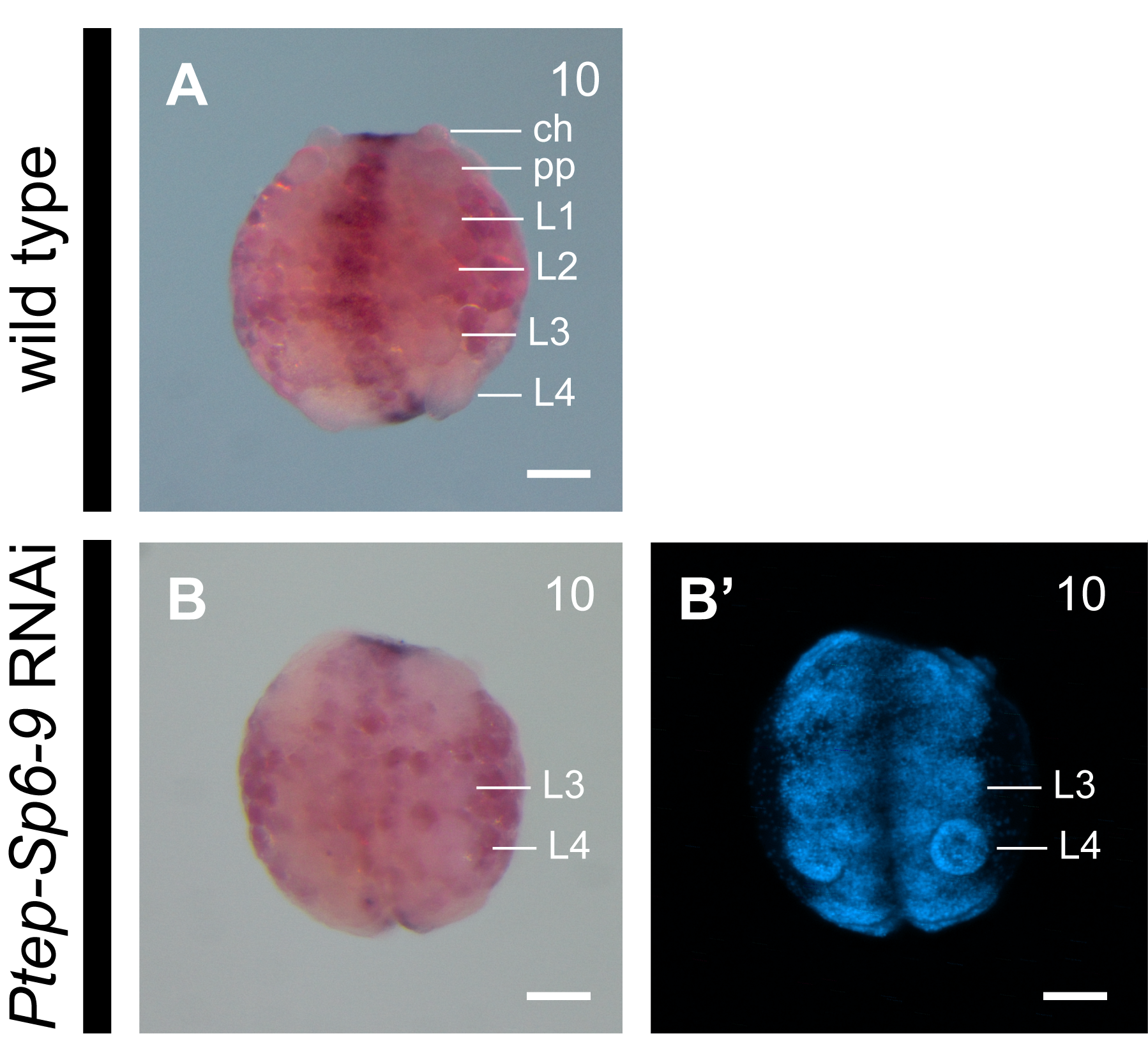

Supplement: S10 Fig — (A) Expression of Ptep-sog is continuous throughout the ventral midline in wild-type embryos. (B) RNAi against Ptep-Sp6-9 results in a gap segmentation phenotype concomitant with the loss of Ptep-sog expression in the affected regions. (B’) Same embryo as in B, with Hoechst counterstain. Abbreviations as in Fig 2. Scale bar: 100 μm. (TIF) [file pbio.3002771.s019.tif]

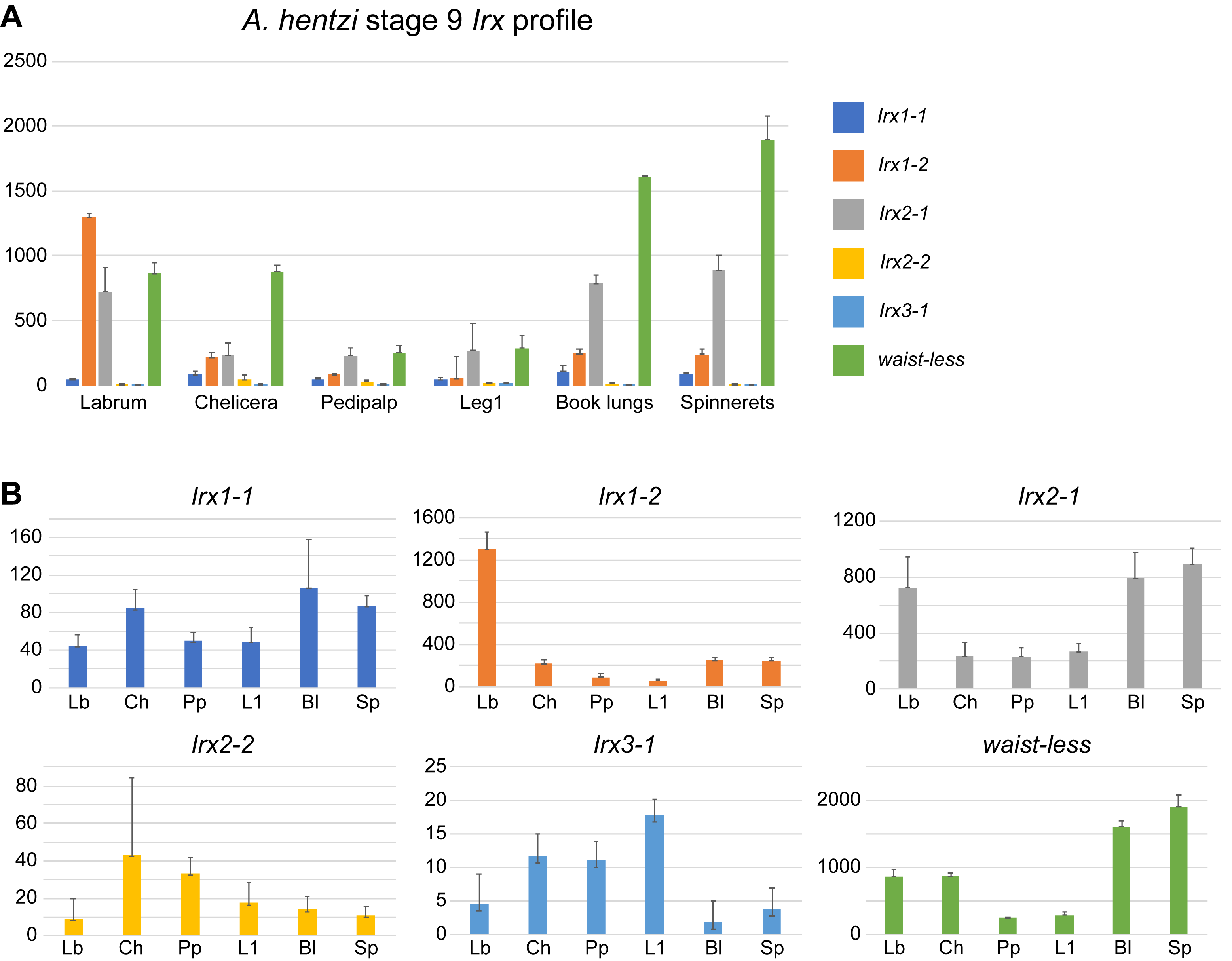

Supplement: S11 Fig — (A) Expression levels for each Iroquois homolog by RNA-seq library (tissue type) in TPM. (B) Individual expression profiles of homologs by tissue type (magnified from panel A) show Ahen-waist-less is not comparably expressed to Ahen-Irx3-1 or other Iroquois homologs. Transcripts of Ahen-waist-less are highly enriched in RNA-seq libraries of opisthosomal tissue, to the exclusion of all prosomal regions sampled. Complete dataset is provided in S2 Data. The data underlying the graphs shown in the figure can be found in S1 Data. bl, book lung; ch, chelicera; lb, labrum; L1, first walking leg; pp, pedipalp; sp, spinnerets. (TIF) [file pbio.3002771.s020.tif]

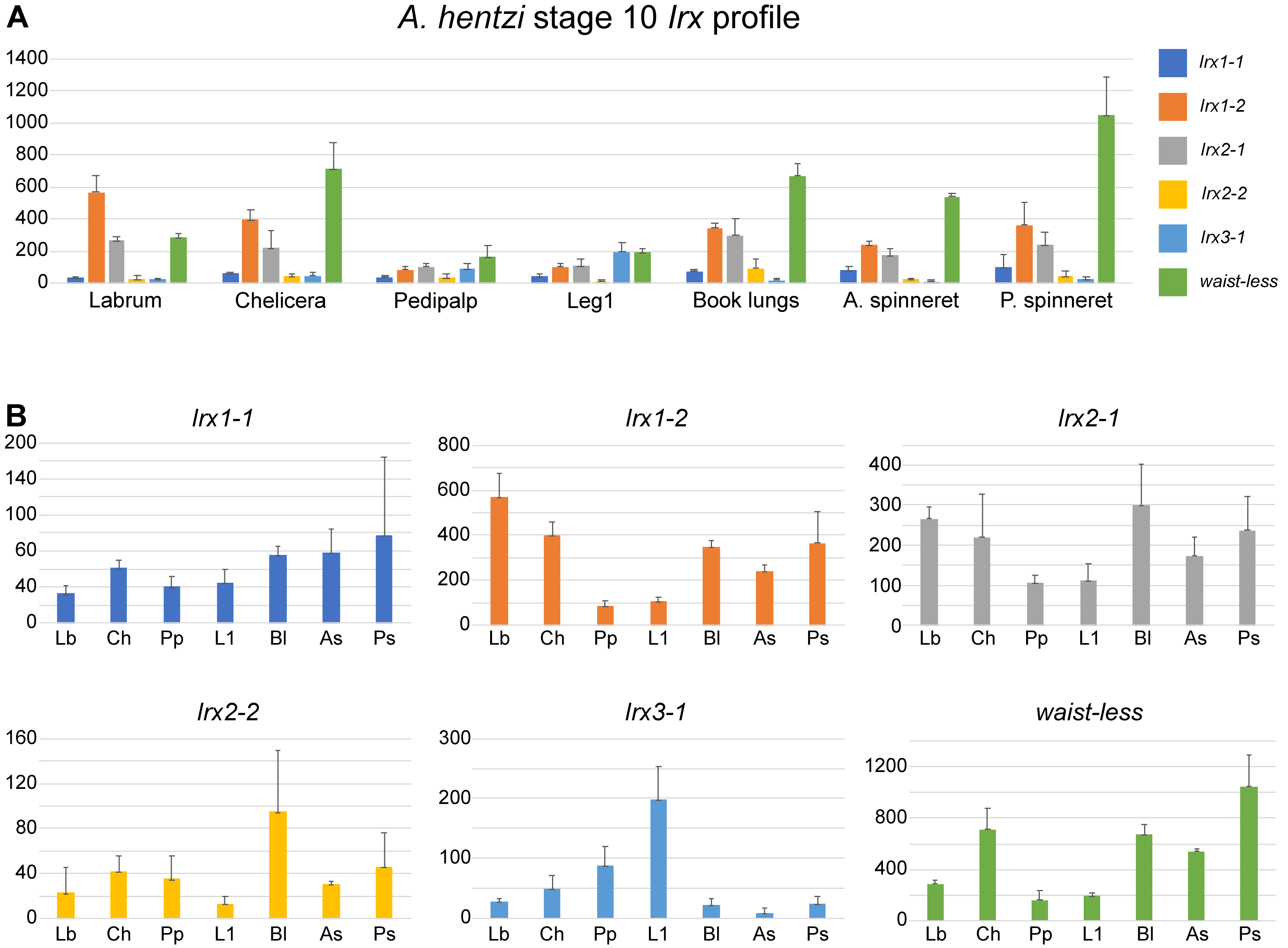

Supplement: S12 Fig — (A) Expression levels for each Iroquois homolog by RNA-seq library (tissue type) in TPM. (B) Individual expression profiles of homologs by tissue type (magnified from panel A) show Ahen-waist-less is not comparably expressed to Ahen-Irx3-1 or other Iroquois homologs. Transcripts of Ahen-waist-less are enriched in RNA-seq libraries of opisthosomal tissue. Complete dataset is provided in S3 Data. The data underlying the graphs shown in the figure can be found in S1 Data. as, anterior spinneret; bl, book lung; ch, chelicera; lb, labrum; L1, first walking leg; pp, pedipalp; ps, posterior spinneret. (TIF) [file pbio.3002771.s021.tif]

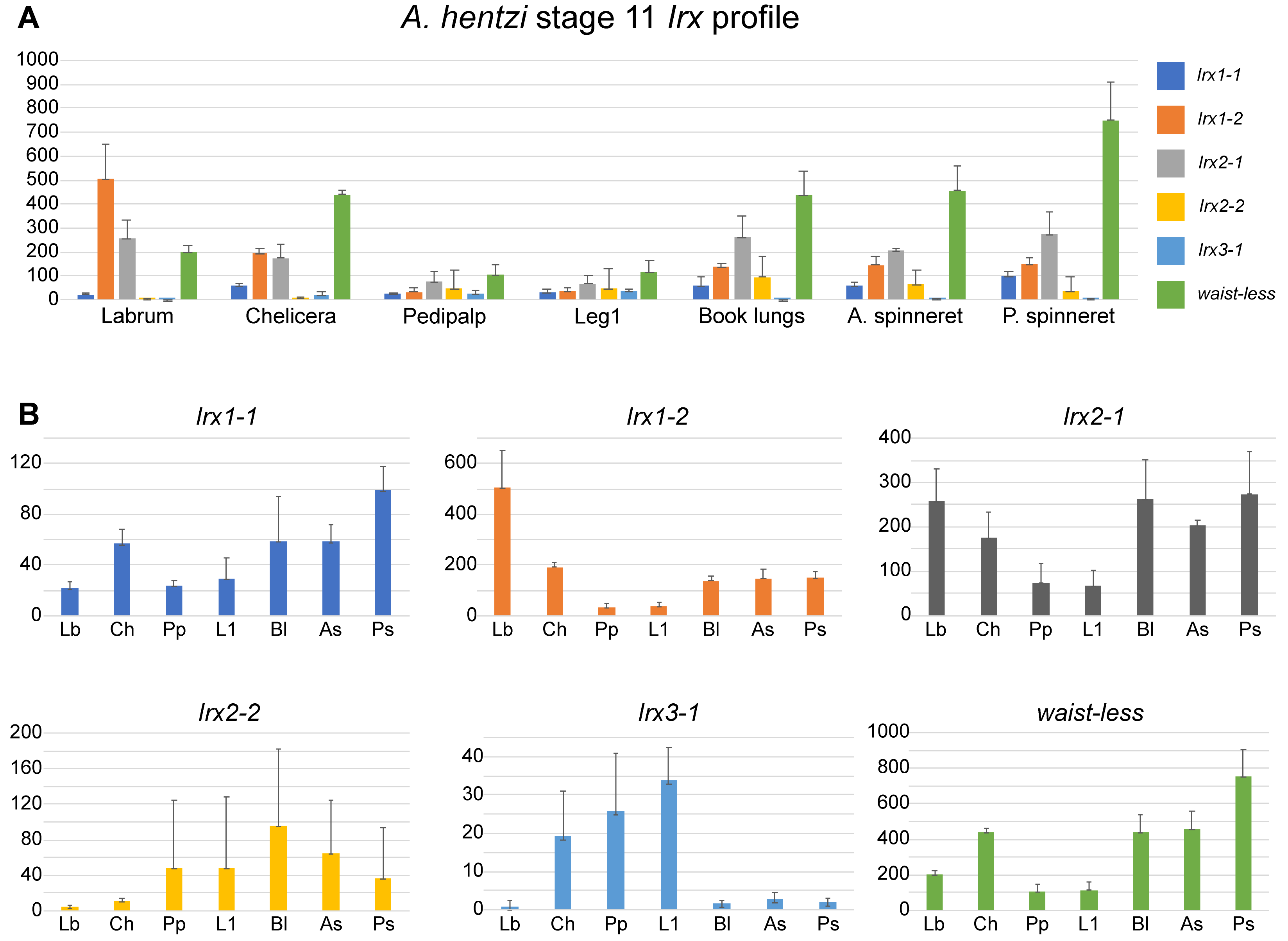

Supplement: S13 Fig — (A) Expression levels for each Iroquois homolog by RNA-seq library (tissue type) in TPM. (B) Individual expression profiles of homologs by tissue type (magnified from panel A) show Ahen-waist-less is not comparably expressed to Ahen-Irx3-1 or other Iroquois homologs. Transcripts of Ahen-waist-less are enriched in RNA-seq libraries of opisthosomal tissue. Complete dataset is provided in S4 Data. The data underlying the graphs shown in the figure can be found in S1 Data. as, anterior spinneret; bl, book lung; ch, chelicera; lb, labrum; L1, first walking leg; pp, pedipalp; ps, posterior spinneret. (TIF) [file pbio.3002771.s022.tif]

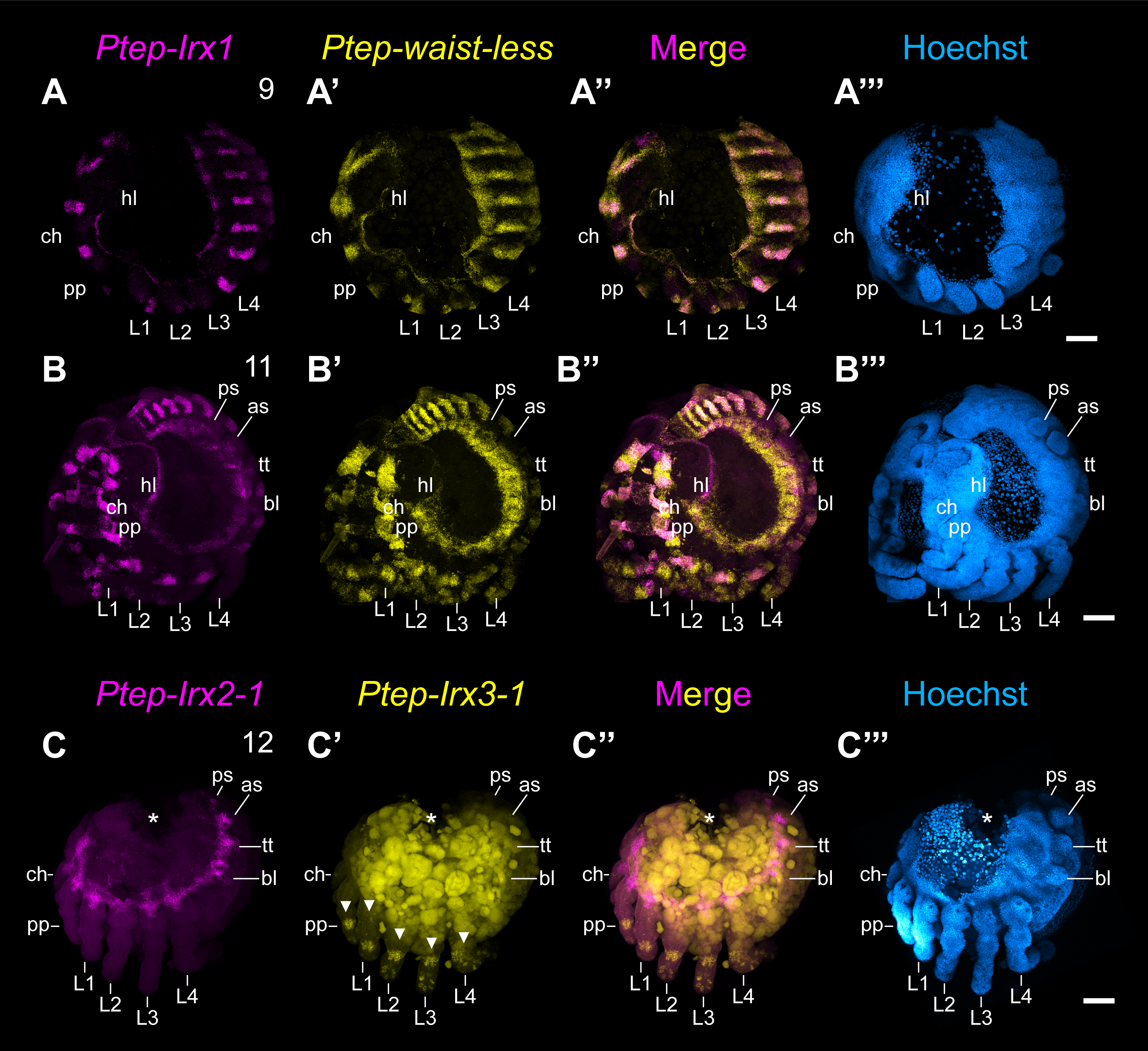

Supplement: S14 Fig — (A-B”’) Ptep-Irx1 and Ptep-waist-less are similarly expressed but have distinct expression domains. Ptep-waist-less is enriched in body wall tissue of the opisthosoma, has a broader expression territory in the head, and has additional expression domains in the legs, as compared to Ptep-Irx1 (A”, B”). (C-C”’) Ptep-Irx2-1 and Ptep-Irx3-1 are not comparably expressed to Ptep-waist-less. Ptep-Irx2-1 is restricted to a uniform band of expression along the lateral margin of the germ band, with slight protrusions into the proximal-most regions of developing appendages (C). Expression of Ptep-Irx3-1 is restricted to 2 non-overlapping expression domains in the developing legs and pedipalps, 1 distal and 1 medial. Ptep-Irx3-1 is notably absent from the chelicera (C’). Asterisks in C-C”’ mark mechanical damage to yolk; note autofluorescence in C’ and C” for Ptep-Irx3-1. Abbreviations as in Fig 2. Scale bars: 100 μm. (TIF) [file pbio.3002771.s023.tif]

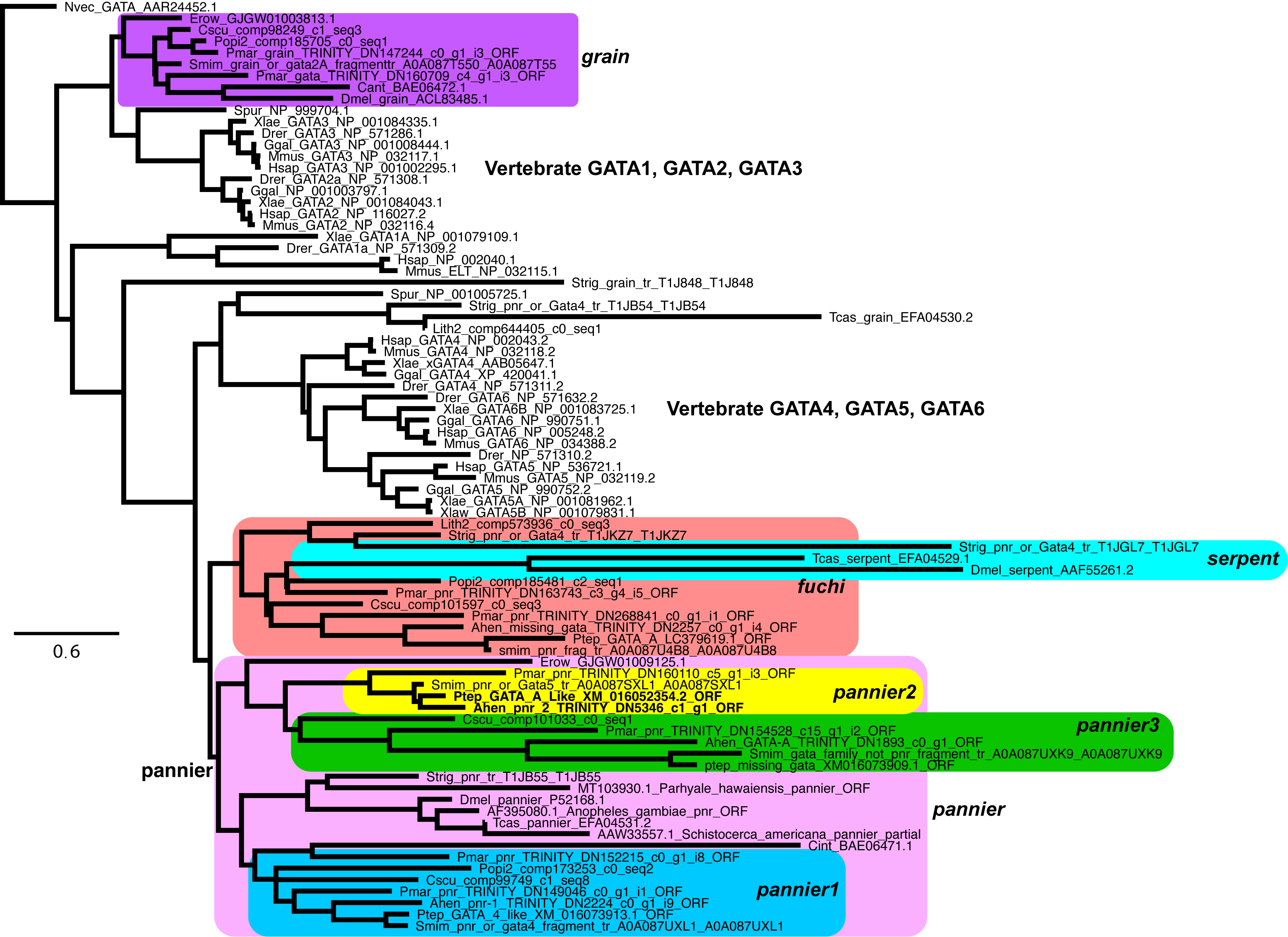

Supplement: S15 Fig — Colors correspond to previously identified paralogs of GATA. Numbers on nodes correspond to bootstrap resampling frequencies. Boldface text indicates pannier2 copies of spiders. (TIF) [file pbio.3002771.s024.tif]

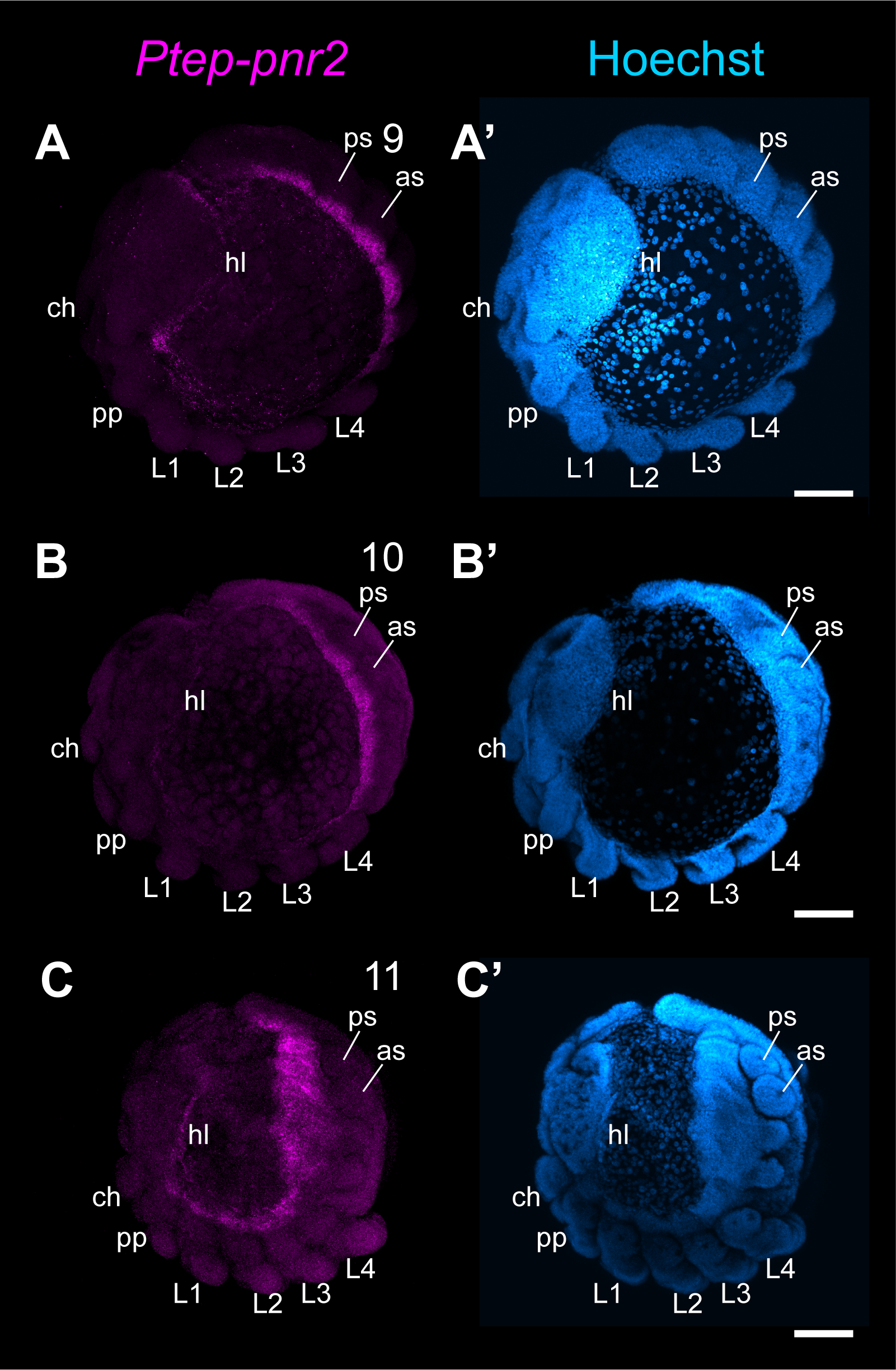

Supplement: S16 Fig — All panels constitute split channel images of the same embryo for Hoechst and HCR in situ hybridization for Ptep-pnr2 (n = 12/12). Abbreviations as in Fig 2. Scale bar: 100 μm. (TIF) [file pbio.3002771.s025.tif]
