# Supplementary material for: A taxon-restricted duplicate of Iroquois3 is required for patterning the spider waist
Source: PLoS Biol. 2024 Aug 29;22(8):e3002771. doi: 10.1371/journal.pbio.3002771 (PMC11361693; doi:10.1371/journal.pbio.3002771)
Supplement: Attachment — Submitted filename: Review.docx [file pbio.3002771.s026.docx]

The paper “A taxon-restricted duplicate of Iroquois3 is required for patterning the spider waist.” Written by Setton and co-workers present data on the differences in transcription between the prosoma and the opisthosoma using transcriptomic analysis. They further identify 16 candidates from the transcriptome data that appear promising in defining the PO axis and the PO boundary. They conduct RNAi and HCR-based *in situ* hybridization experiments and show that *irx4* is involved in the regulation of the dorsoventral axis in the precise location of the PO boundary, which results in a waist-less loss of function phenotype. Furthermore, the analysis of markers post RNAi shows that the components of the DV axis regulation such as *sog* and *pnr* homologs are affected in the region.

The paper is well-written and presents interesting data on the development of spiders. This is promising given the technical challenges in this type of organism, where experimental tools are not as well developed as in model organisms.

In my opinion, this paper is fit for publication with minor corrections in the text.

Minor revisions needed:

One of the gene names is not consistent and is interchangeably swapped between *irx4, ptep-irx4,* and *waist-less*. The authors should clarify this early on in the text and use the same name in the main text as well as supplementary tables.

lines 157, 159, and 177: The usage of the words ‘exemplars’ is not conventional and could confuse a wider audience.

lines 87, 114: Like my previous comment, the usage of the word ‘triangulate’ appears wrong. The term could be replaced by a more widely used term.

Lines 313, and 315: The reference to the figure is wrong, there are only As and Bs in Figure 5.

Line 578: The sentence should read, “The negative controls were injected….”

Lines 318-320: Could be revised to something similar in lines 387-388. The term territory-specific makes the phenotype more understandable.
