# Supplementary material for: A taxon-restricted duplicate of Iroquois3 is required for patterning the spider waist
Source: PLoS Biol. 2024 Aug 29;22(8):e3002771. doi: 10.1371/journal.pbio.3002771 (PMC11361693; doi:10.1371/journal.pbio.3002771)
Supplement: Attachment — Submitted filename: Responses to reviewers.docx [file pbio.3002771.s027.docx]

**Responses to reviewers**

We thank both reviewers and the editor for their patience during our revisions. Due to the departure of the first author after completing her graduate program, this study has progressed slowly for the past eight months. Our revisionary efforts have focused on rewriting the manuscript for clarity and on generating new expression data for *waist-less* in early spider embryonic stages, as requested by the first reviewer.

We also spent considerable time on the first reviewer’s suggestion that *waist-less* may play a role as a gap segmentation gene, rather than a localized DV patterning gene. Our previous draft was predicated on the assumption that disruption of *sog* expression reflected a DV phenotype. To address the reviewer’s point, we examined earlier expression of *waist-less* and corroborated its expression in early (pre-germ band) stages as reflective of a segmentation function. In addition, to test our interpretation of DV versus gap phenotype, we performed RNAi against well-characterized gap genes (*hunchback* and *Sp6-9*) and assayed these for *sog*. We found that segments disrupted by RNAi against these gap genes (i.e., L1 and L2) also exhibited diminution of *sog* expression. These experiments suggest that localized interruption of *sog* expression is consistent with a gap segmentation phenotype, contrary to our previous interpretation.

We have therefore rewritten our manuscript to reflect that *waist-less* is required for patterning the prosoma-opisthosoma boundary and most likely acts as a trunk gap gene in spiders.

**Reviewer 1:

Major points:
1. Further information regarding the tissue-specific RNA-seq methods and subsequent DGE analyses should be provided. Was total RNA extracted from the main body, lateral-dorsal parts of the body, or appendages? (p5, l09 or p20, 490.) What is the expression pattern of the identified genes in the embryo? Were they expressed region specifically? Brief statements summarizing the DGE analysis are needed.**

Response: We appreciate the reviewer’s attention to detail and have provided additional information regarding the tissues from which total RNA was extracted. We have also added new supplementary data detailing expression levels of the selected genes across stages. Many of the genes identified in our DGE analyses had well-known, region-specific expression patterns, such as the Hox genes. However, we do not report the expression pattern of all identified genes, for two reasons. First, during the lifetime of this study (2018 to present), reporting of expression patterns in *P. tepidariorum* has been greatly accelerated by the combination of single-cell and single-nucleus sequencing and the advent of fluorescent detection techniques like hybridization chain reaction (HCR). By contrast, we struggled to obtain clear signal-to-noise ratios in colorimetric in situs of *P. tepidariorum* for many of our genes. The second and more immediate reason why we focused more on RNAi than expression is that—in our hands—RNAi has proven highly variable in *P. tepidariorum*, both with respect of proportion of genes that yield phenotypes and with respect to number of embryos that exhibit phenotypes in successful experiments. This experience is reflected in a handful of published works. As examples, Akiyama-Oda and Oda (2006) showed that penetrance varies markedly across injected females in a single experiment using maternal RNAi. Similarly, Pechmann et al. (2017) showed that only two out of 35 genes screened from bioinformatic predictions yielded a phenotype using the same RNAi technique.

In our lab, we have trialed RNAi for dozens of genes (e.g., Hox genes; segmentation genes; Wnts; leg patterning genes; spinneret-enriched genes), but recovered few experiments with robust phenotypic outcomes, despite promising gene expression patterns and well-characterized in situ surveys (e.g., *extradenticle* copies; *homothorax* copies; *wingless*). For this reason, this study prioritized the RNAi screen over the expression surveys; we wanted to generate new phenotypes first and foremost.

**With respect to the waist-less gene, the expression does not seem clearly different among the segments of the opisthosoma and the prosoma in the stained embryos (fig. S3). In the RNA-seq data (fig. S11-13), the expression levels appear to be correlated with the expression in the appendages. Further comments and discussion on these points are needed.**

Response: Kindly examine Figure 1B-1D. We take the reviewer’s point that the difference in expression pattern in subtle, but higher expression of *waist-less* in the opisthosomal segments is visible in these embryos. The overstaining applied to the embryos in Figure S3 to generate greater contrast in merged images diminishes this effect, but even these embryos are visibly darker in their opisthosomal segments.

**2. The expression profiles of Irx genes in A. hentzi (fig. S11-13) are mentioned only in the Discussion section. This data should be stated much earlier in the Results section. Similar profiles for the 16 priority genes are informative and should be presented if possible.**

Response: We have followed the reviewer’s request and presented these (and other new) expression profiles in the Results.

**3. The authors concluded that waist-less played a role in dorso-ventral patterning. However, the phenotypes of the waist-less knockdown embryos were analyzed only in the late stages when morphological abnormalities were noticeable. It is possible that the described phenotypes are secondary effects following the defects in germ band morphology. Furthermore, the waist-less gene is expressed in the embryo at late stage 5, as shown in Sci. Adv. 2020; 6, eaba7261 (Data file S5-7, aug3.g8295). The position of early expression along the AP axis seems to be near the boundary between the prosoma and opisthosoma. Knockdown embryos should be examined for the earliest defects; re-examining gap phenotypes and dorso-ventral patterning defects in earlier embryos would be interesting. Additionally, how is the AP and DV pattern in the embryo shown in Fig. S4?**

Response: We thank the reviewer for this suggestion and for pushing our thinking. As requested, we examined *waist-less* expression at earlier developmental stages and we indeed observed segmentation gene-like expression patterns in stages preceding the formation of the germ band. We added the citation to the late stage 5 expression, corroborated that expression in this work, and examined subsequent stages over several hours to follow the dynamics of *waist-less*.

While we did try to understand the effects of *waist-less* RNAi in earlier stages in the past several months, we struggled to obtain a large number of embryos that exhibited a phenotype before the formation of the limb buds. For this reason, we were not able to describe early defects of the germ band, nor the regulatory interaction of *waist-less* and other gap genes like *hunchback* and *Sp6-9*; we present these avenues of inquiry for future studies of spider segmentation.

**4. Although pannier2 gene was included in the list of 16 priority genes for functional screening (Table S1), it was presented only in the last part without mentioning the screening. Consistent statements are required to address this point.**

Response: We have added reference to *pannier2* in the initial discussion of the RNAi screen. We have tried to be as transparent as possible about why *pannier2* was not investigated to the same degree as *waist-less*.

**5. Very beautiful pictures showing the expression of Irx genes of P. opi and P. tep were mentioned only in the Discussion section. The data should be stated in the Results section.**

Response: As requested, we have rewritten the manuscript to present these datasets in the Results.

**Minor points:
1. The reasons for the selection of the 16 genes (and unselection of other genes) for functional screening (p5, l17-120) should be shown concretely, for example using tables that display quantified expression levels in each sample.**

Response: We have added additional text to better detail the candidate selection process. We have also included new supplementary figures providing the reads recovered per sample, for each gene included in the RNAi screen. Please note that to some degree, various genes were not selected simply due to time constraints.

**2. The criteria for assessing phenotypes during the screening should be included (p6, 138). Were the embryos stained for the expression of certain genes or examined morphologically? At which stages were the embryos examined?**

Response: The text has been amended to clarify how phenotypes were identified and across which stages.

**3. As in p6, 144, the names of genes have been confused. It would be helpful to attach LOC numbers in the NCBI database for listed genes.**

Response: We thank the reviewer for this observation. We have included a new supplementary table (Table S2) with gene IDs for candidate transcripts. This document provides the Trinity headers for each gene for both publicly available *A. hentzi* transcriptomes and orthologous *P. tepidariorum* sequences identified at the time of the RNAi screen. The *P. tepidariorum* orthologs have both the NCBI LOC numbers and genbank accession numbers provided in this document. In order to preserve transparency and clearly convey the orthologous genes identified in *P. tepidariorum*, the LOC and genbank numbers for the house spider are presented in this document under the identifiers they were associated with at the time of gene orthology searches for the RNAi candidate genes in *P. tepidariorum*). We have additionally corrected Table S1 to provide the *P. tepidariorum* LOC number for the uncharacterized transcripts; the original submission erroneously listed the *A. hentzi* Trinity identifier as the LOC number.

**4. The name Irx4 may not be correct (p8, 185). Verify and revise as necessary.**

Response: We have corrected the confusion regarding nomenclature.

**5. I understand that the staining shown in Fig. 3 and 4 was performed using mixed probes. It is better to include a note on this and briefly explain the expression patterns of each gene.**

Response: We now explain the individual expression patterns in the text and provide citations corresponding to those data.

**6. Fig. 5E (p13, 313) and Fig. 5F. 5G (p13, 315) need to be rechecked and revise for accuracy.**

Response: We regret the incorrect figure references. These errors have been corrected.

**7. Regarding the phenotype of pnr2 knockdown embryo, the graph (Fig. 6E) showed that only a small percentage of embryos exhibited this phenotype. How and when were the embryos counted? Did embryos without this phenotype exhibit decreased pnr2 expression?**

Response: We have clarified the criteria for phenotypic screening of both *waist-less* and *pnr2* RNAi embryos. We did not observe visibly reduced *pnr2* expression in wild type embryos; as with *waist-less*, the degree of phenotypic severity was correlated with expression level of the targeted gene.

**8. I cannot understand Fig. 7. Do the illustrations on the right and left show differences in the developmental stages? This legend should be rewritten with more detail.**

Response: We regret the lack of clarity in the previous version of this figure. We have designed a new summary figure to convey the conclusion of this study more clearly.

**9. How many times was the dsRNA injection performed (p23, p24)?**

Response: We have clarified that four injections were performed over an eight-day interval.


**Rev. 2: Ab. Matteen Rafiqi - note that this reviewer has signed his review
The paper "A taxon-restricted duplicate of Iroquois3 is required for patterning the spider waist." Written by Setton and co-workers present data on the differences in transcription between the prosoma and the opisthosoma using transcriptomic analysis. They further identify 16 candidates from the transcriptome data that appear promising in defining the PO axis and the PO boundary. They conduct RNAi and HCR-based in situ hybridization experiments and show that irx4 is involved in the regulation of the dorsoventral axis in the precise location of the PO boundary, which results in a waist-less loss of function phenotype. Furthermore, the analysis of markers post RNAi shows that the components of the DV axis regulation such as sog and pnr homologs are affected in the region.

The paper is well-written and presents interesting data on the development of spiders. This is promising given the technical challenges in this type of organism, where experimental tools are not as well developed as in model organisms.
In my opinion, this paper is fit for publication with minor corrections in the text.**

Response: We thank this reviewer for their generous evaluation of the work.

**Minor revisions needed:**

**One of the gene names is not consistent and is interchangeably swapped between irx4, ptep-irx4, and waist-less. The authors should clarify this early on in the text and use the same name in the main text as well as supplementary tables.**

Response: We have adopted a consistent nomenclature throughout the study and clarified the history of this gene’s naming conventions in the text.

**lines 157, 159, and 177: The usage of the words 'exemplars' is not conventional and could confuse a wider audience.**

Response: We have substituted the use of “exemplars” with “representatives”

**lines 87, 114: Like my previous comment, the usage of the word 'triangulate' appears wrong. The term could be replaced by a more widely used term.**

Response: We have substituted the use of “triangulate” with “identify”.

**Lines 313, and 315: The reference to the figure is wrong, there are only As and Bs in Figure 5.**

Response: This has been corrected.

**Line 578: The sentence should read, "The negative controls were injected…."**

Response: This has been corrected.

**Lines 318-320: Could be revised to something similar in lines 387-388. The term territory-specific makes the phenotype more understandable.**

Response: Please see responses to reviewer 1. Due to extensive rewriting, this passage no longer appears in the current draft.
